# Magnesium Anode Protection by an Organic Artificial Solid Electrolyte Interphase for Magnesium-Sulfur Batteries

**DOI:** 10.1021/acsami.3c07223

**Published:** 2023-06-30

**Authors:** Joachim Häcker, Tobias Rommel, Pia Lange, Zhirong Zhao-Karger, Tobias Morawietz, Indro Biswas, Norbert Wagner, Maryam Nojabaee, K. Andreas Friedrich

**Affiliations:** † Institute of Engineering Thermodynamics, German Aerospace Center (DLR), Pfaffenwaldring 38-40, 70569 Stuttgart, Germany; ‡ Institute of Inorganic Chemistry, University of Stuttgart, Pfaffenwaldring 55, 70569 Stuttgart, Germany; § Helmholtz Institute Ulm (HIU) Electrochemical Energy Storage, Helmholtzstrasse 11, 89081 Ulm, Germany; ∥ Faculty of Science, Energy and Building Services, Esslingen University of Applied Sciences, Kanalstraße 33, 73728 Esslingen am Neckar, Germany; ⊥ Institute of Building Energetics, Thermal Engineering and Energy Storage (IGTE), University of Stuttgart, Pfaffenwaldring 6, 70569 Stuttgart, Germany

**Keywords:** magnesium−sulfur battery, magnesium anode, ionomers, artificial solid electrolyte interphase, electrochemical impedance spectroscopy, coating techniques

## Abstract

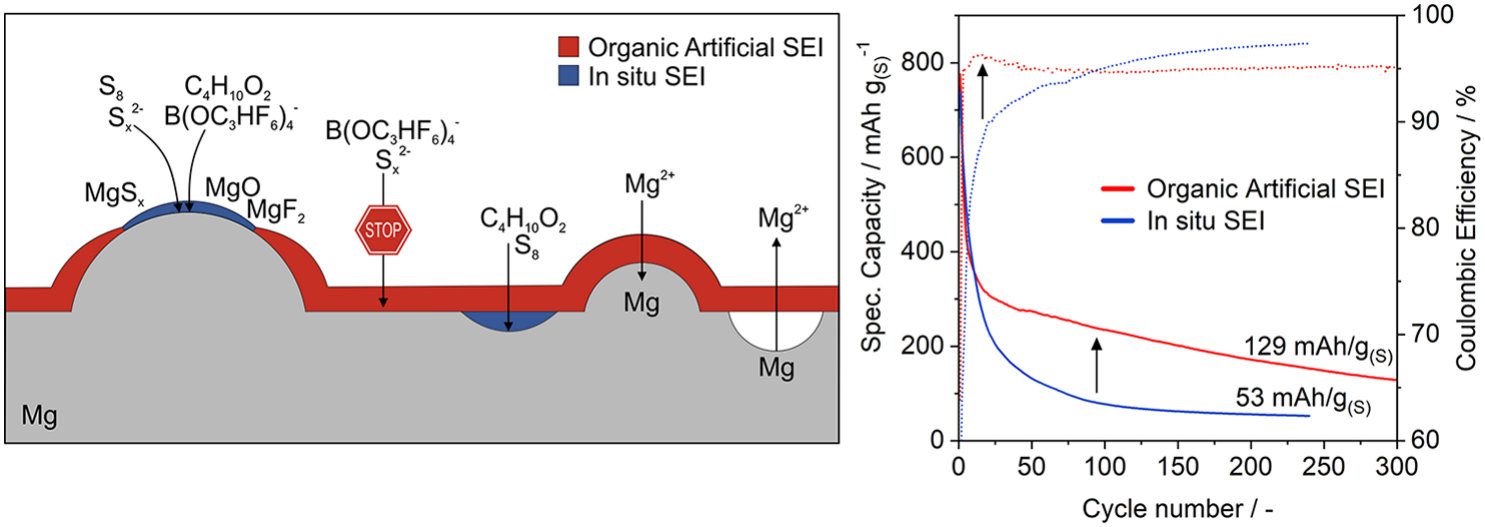

In the search for post-lithium battery systems, magnesium–sulfur batteries have attracted research attention in recent years due to their high potential energy density, raw material abundance, and low cost. Despite significant progress, the system still lacks cycling stability mainly associated with the ongoing parasitic reduction of sulfur at the anode surface, resulting in the loss of active materials and passivating surface layer formation on the anode. In addition to sulfur retention approaches on the cathode side, the protection of the reductive anode surface by an artificial solid electrolyte interphase (SEI) represents a promising approach, which contrarily does not impede the sulfur cathode kinetics. In this study, an organic coating approach based on ionomers and polymers is pursued to combine the desired properties of mechanical flexibility and high ionic conductivity while enabling a facile and energy-efficient preparation. Despite exhibiting higher polarization overpotentials in Mg–Mg cells, the charge overpotential in Mg–S cells was decreased by the coated anodes with the initial Coulombic efficiency being significantly increased. Consequently, the discharge capacity after 300 cycles applying an Aquivion/PVDF-coated Mg anode was twice that of a pristine Mg anode, indicating effective polysulfide repulsion from the Mg surface by the artificial SEI. This was backed by operando imaging during long-term OCV revealing a non-colored separator, i.e. mitigated self-discharge. While SEM, AFM, IR and XPS were applied to gain further insights into the surface morphology and composition, scalable coating techniques were investigated in addition to ensure practical relevance. Remarkably therein, the Mg anode preparation and all surface coatings were prepared under ambient conditions, which facilitates future electrode and cell assembly. Overall, this study highlights the important role of Mg anode coatings to improve the electrochemical performance of magnesium–sulfur batteries.

## 1 Introduction

In recent years, the magnesium–sulfur battery has attracted attention as a post-lithium system due to its high theoretical volumetric energy density, potential low-cost raw materials, abundance, and improved safety. Large progress has been achieved especially regarding efficient electrolytes; however, the system still features severe self-discharge and poor long-term cycling stability generally assigned to the polysulfide dissolution and shuttle.^1,2^ Besides the sulfur cathode and its abilities to retain polysulfides, the anode plays a major role in this context since the undesired polysulfide reduction takes place at the magnesium surface. Moreover, the sulfur species contribute to the Mg surface layer formation, the solid electrolyte interphase (SEI),^3,4^ which might result in large overpotentials or complete passivation.

Although three-dimensionally structured anodes like Mg powder pellets,^5^ Mg_3_Bi_2_,^6^ microporous graphitic carbon nanosubstrates,^7^ or N-/O-doped carbon cloths^8^ can decrease the tendency to electrolyte reduction and nucleation overpotential by providing a higher surface area and thus a lower current density, the anode is still susceptible to reactions with sulfur species. In a modeling approach, Richter et al. demonstrated that the implementation of an ex situ surface coating, a so-called artificial SEI, is superior to sulfur retention approaches at the cathode (e.g., sulfur infiltration in micropores^9^) as the already sluggish redox kinetics of magnesium–sulfur batteries are otherwise further suppressed.^10^

Many studies follow an in situ artificial SEI approach by utilizing electrolyte additives like MgCl_2_,^11^ I_2_,^12,13^ GeCl_4_,^14^ Pyr_14_^+^,^15^ Al(O_2_C_2_(CF_3_)_4_)_2_^–^,^16^ Mg(BH_4_)_2_,^17^ Bi(OTf)_3_,^18^ or (CH_2_CO)_2_O^19^ or applying a hybrid electrolyte incorporating high concentrations of LiBH_4_,^20,21^ LiCl,^21^ LiBr,^22^ LiI,^23^ LiTFSI,^24,25^ Li[B(hfip)_4_],^26^ or LiCF_3_SO_3_.^27^ However, an in situ SEI formation is rather uncontrolled in composition, homogeneity, and thickness, and an ongoing salt and additive consumption might occur. For that reason, an ex situ approach via practical coating techniques might be superior and the preparation methods are manifold: Li et al. report about a MgF_2_ layer prepared by sonification of Mg in a hydrofluoric acid solution,^28^ Wang et al. realized fast charge transfer kinetics by an electrodeposited bismuth layer,^29^ Zhang et al. constructed an artificial SEI by high-temperature displacement reaction of CuCl_2_ and Mg,^30^ and Sahadeo et al. investigated atomic-layer-deposited Al_2_O_3_,^31^ while Park et al. followed a chemisorption approach to form MgSO_3_ on Mg in a SO_2_ gas atmosphere.^32^ Other studies simply wet the Mg surface, e.g., with tin halides/G1,^33^ BiCl_3_ + Bi(CF_3_SO_3_)_3_,^34,35^ SiCl_4_/G1,^36^ or liquid gallium^37,38^ to result in a Sn-based coating, Bi-based metal–alloy hybrid layer, Si–O and Si–C-reinforced organic matrix, or Mg_2_Ga_5_-alloyed surface, respectively.

Several studies demonstrate that an artificial interphase enables the use of alternative electrolyte solvents, which are currently restricted to ethers like glymes, dioxolane, or tetrahydrofuran. The application of carbonate-based electrolytes, which are otherwise incompatible with Mg metal,^39^ is enabled by a protective layer composed of thermal-cyclized PAN and magnesium triflate,^40^ by immersing Mg metal in a LiTFSI + AlCl_3_/G4 solution^41^ or by applying an amorphous structure composed of a PVDF-HFP backbone with TFSI-grafted anions.^42^ Thus, an artificial SEI aims to solve several problems at once. Above all, it shall hinder the contact of sulfur and electrolyte species with the Mg surface, which not only excludes ongoing parasitic reactions and therefore active material loss and electrolyte overconsumption but also widens the electrolyte solvent choice.

The majority of the reported coatings are composed of inorganic materials like halides (MgF_2_, MgCl_2_, MgBr_2_, or MgI_2_), Mg(BH_4_)_2_, MgSO_3_, or Mg alloys, which offer a high electrochemical stability.^43,44^ However, they are prone to breakage or delamination upon cycling due to the morphology changes of the anode surface. Furthermore, the Mg^2+^ conductivity might be rather low due to the high intrinsic Mg^2+^ migration barrier of the crystalline bulk material.^44^ In contrast, organic coatings like PAN-Mg(OTf)_2_,^40^ TFSI@PVDF-HFP,^42^ a-MgCl_2_@polymer,^45^ and MOFs^46^ or gel-like polymer electrolytes like PTHF-Mg(B(hfip)_4_)_2_,^47^ FTGB-MgCl,^48^ PEO-Mg(BH_4_)_2_-MgO,^49^ PVA-Mg(OTf)_2_,^50^ and TEP-Mg(ClO_4_)_2_,^51^ feature nongranular, mainly amorphous microstructure and tailorable Mg^2+^ conductivity by incorporation of inorganic additives (hybrid approach). As the latter may diffuse and agglomerate, ionomers comprising covalently bound functional groups like PEGDMA–STFSI,^52,53^ which enable ion conductivity might be superior. In addition, polymeric coatings can be easily prepared and provide large tolerance to morphology changes due to their intrinsic elasticity. One major drawback, however, is their (electro)chemical stability being lower compared to inorganic materials.

In this study, an organic artificial SEI approach and the techniques of spin-coating, dip-coating, and tape casting were utilized. Two ionomers, namely, a sulfonated tetrafluoroethylene-based copolymer (Aquivion) and sulfonated poly(etheretherketone) (SPEEK) mixed with poly(vinylidene fluoride) (PVDF) to combine ionic conductivity and mechanical stability were selected. In comparison, a coating based on PAN and Mg triflate analogous to previous studies was applied.^40^ Extensive analysis via SEM-EDX, AFM, IR and XPS reveals insights into the surface morphology and composition, while the electrochemical performancewas investigated by polarization experiments, electrochemical impedance spectroscopy (EIS) and galvanostatic cycling in Mg–Mg and Mg-S cells, respectively. Utilizing Mg-Aquivion/PVDF and Mg-PAN anodes, the discharge capacity of Mg–S cells after 150 cycles was significantly increased compared to a pristine Mg foil (201/126 vs 69 mAh/g_(S)_). Furthermore, the Coulombic efficiency is enhanced, reflecting the obviation of parasitic reactions—namely, electrolytes and sulfur reduction.

## 2 Experimental Section

### 2.1 Artificial SEI on Magnesium Metal

2.5 wt % solutions of Aquivion (PW 79S, *M*_w_ = 790 g/mol, Sigma-Aldrich), sulfonated poly(etheretherketone) (SPEEK, Fumatech GmbH), and poly(vinylidene fluoride) (PVDF, Solef 5301, Solvay) in dimethylacetamide (DMAc, 99.5%, Acros Organics) were prepared by stirring at 500 rpm. While the ionomers were easily soluble at 25 °C, the latter was heated to 100 °C and stirred over night for complete dissolution. Subsequently, the ionomer solutions were mixed with the PVDF solution in a volumetric ratio of 1:1 and used as coating inks (Aquivion/PVDF and SPEEK/PVDF). Additionally, a 3.4 wt % solution of poly(acrylonitrile) (PAN, 99.5%, Goodfellow) in dimethylformamide (DMF, 99.5% Acros Organics) was prepared with magnesium trifluoromethanesulfonate (Mg triflate, 98%, Alfa Aesar) being added to the solution to result in a PAN/Mg triflate ratio of 10:3—analogous to Son et al.^40^

The preparation of the ionomeric artificial SEI on Mg was done via dynamic spin-coating under ambient conditions in the fume hood (Figure S1). Beforehand, magnesium metal (100 μm, Gelon) was scraped with a glass plate to remove the surface oxide layer and cut into 18 mm electrodes. Subsequently, the Mg substrate was placed in a SPIN 150i spin coater (SPS), and 150 μL of coating solution was injected on the magnesium surface at a constant spin speed of 1000 rpm. After spinning for 30 s and drying under ambient conditions, the coated Mg foil was transferred into the glovebox for cell assembly. In the case of the PAN-coating, an additional heating step at 250 °C/vacuum was applied to initiate the ring polymerization to c-PAN.

To enable the coating of larger Mg substrates, scalable techniques were utilized in a first attempt. Dip-coating was applied with the coating solution being filled in a glass dish, and the 18 mm Mg foil pulled through to wet the surface. For tape casting, the Mg foil was fixed on a vacuum table (Zehntner ZAA 2300) and coated using 10 μL/cm^2^ (50 μm wet thickness, Zehntner ZUA 2000). In both cases, the coatings were subsequently dried under ambient conditions.

### 2.2 Cathode and Electrolyte Preparation

To identify the benefit of an artificial SEI in early stages of cycling, a cathode approach with mechanically rather than melt-infiltrated sulfur was chosen. Furthermore, an aqueous slurry was utilized to establish an environmentally friendly and energy-efficient preparation route, which is depicted in detail in a previous study.^2^ In brief, sulfur (99.5%, Alfa Aesar) and Ketjenblack EC 600-JD (Akzo Nobel) were ball-milled at 500 rpm for 15 min in a mass ratio of 5:4. Subsequently, aqueous carboxymethyl cellulose solution (3.7 wt % CMC, Walocel CRT 2000 PA, Dow Wolff) and styrene-butadiene rubber solution (40.4 wt % SBR, JSR TRD 102A, JSR Micro) were added to the ball-milled powder. After stirring, a well-dispersed slurry was achieved, which was doctor-bladed on carbon-coated aluminum foil (22 μm) and dried at ambient conditions. Finally, a cathode with 50:40:10 wt % S/KB/CMC-SBR (CMC/SBR 1:2) and approximately 1.0 mg_(S)_/cm^2^ results.

The synthesis of magnesium hexafluoroisopropyloxy borate (Mg[B(hfip)_4_]_2_) was executed according to previous studies,^54^ and the salt was thoroughly dried at elevated temperatures from RT to 60 °C for 15 h in vacuum (0.1 Pa) before use. Subsequently, 1 mmol Mg[B(hfip)_4_]_2_ was dissolved in 5 mL of dimethoxyethane (G1, monoglyme, 99.5%, <10 ppm H_2_O, Acros Organics), stirred over night, and purified with a PTFE syringe filter to result in a 0.2 M electrolyte.

### 2.3 Cell Assembly and Characterization

The electrochemical characterization of the artificial SEI was done in Mg–Mg and Mg–S cells. Therefore, three-electrode ECC PAT-Core cells (El-Cell) with a magnesium metal reference ring, two separator layers of glass fiber (GF/C, Whatman), 18 mm electrodes, and 200 μL of electrolyte were used (Figure S2). The cell assembly was done in an argon-filled glovebox (O_2_ < 1 ppm, H_2_O < 3 ppm).

The symmetrical Mg–Mg cells were investigated with a testing procedure including an initial 50 h rest at OCV and subsequent stripping/plating at different current densities (0.1, 0.2, 0.5, 1.0, and 0.1 mA cm^–2^)—each for 10 cycles with subsequent 10 h OCV. Electrochemical impedance spectra were recorded vs Mg reference electrode in specific time intervals during OCV (potentiostatic, 5 mV amplitude) and at the end of selected polarization steps (galvanostatic, *I*_EIS_ = *I*_pol_, 5 mV amplitude). The EIS measurements were performed with a ZENNIUM and IM6 potentiostat (Zahner) in a frequency range of 100 mHz to 300 kHz. Additionally, long-term polarization at 0.1 and 1.0 mA cm^−2^ without EIS measurements was executed. Mg–S cells were cycled at 25 °C with a *C*/10 rate (167.5 mA/g_(S)_) in a voltage range of 0.05–3 V with an initial 1 h OCV after cell assembly.

Atomic force microscopy (AFM) was performed using a Bruker Icon XR AFM in tapping PeakForce-QNM mode with RTESP-300 tips (Bruker, *k* = 40 N/m, 8 nm tip radius). 512 pixels were recorded at a scan rate below 1 Hz. The image size was 10 × 10, 3 × 3, and 1 × 1 μm^2^ as well as several large area scans up to the maximum scan size of the scanner (95 × 95 μm^2^). The samples were glued to 15 mm AFM stainless steel disks using double sided tape.

Depth profiling via X-ray photoelectron spectroscopy (XPS) was conducted on a spectrometer with a monochromatic Al Kα X-ray source (excitation energy: 1486.74 eV) and a hemispherical electron analyzer (Thermo Fisher Scientific ESCALAB Xi+) at a base pressure of ∼2 × 10^–10^ mbar. Samples were etched under an Ar^+^ ion beam of 2000 eV beam energy applied to a square-shaped sample area of 2 mm^2^, resulting in an average sample current of ∼0.9 μA. Evaluation and quantification was carried out by numerically fitting of convoluted Gaussian/Lorentzian profiles.^55^

Scanning electron microscopy–energy-dispersive X-ray (SEM-EDX) analysis was done on an ULTRA Plus microscope (Zeiss) with a QUANTAX 400 spectrometer and XFlash detector 5010 (Bruker). Infrared spectra were gained applying a Nicolet iS5 spectrometer (Thermo Fisher Scientific).

## 3 Results and Discussion

### 3.1 Artificial SEI

**Figure 1:**
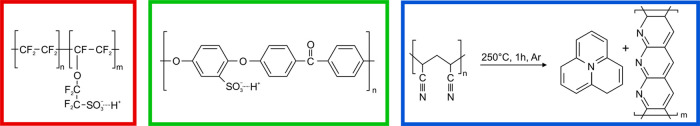
Chemical structure of Aquivion (red), SPEEK (green), and c-PAN (blue). Both ionomers comprise one sulfonyl functional group per repetition unit but exhibit different ion exchange capacity values: IEC (Aquivion) = 1.3 mequiv/g, IEC (SPEEK) = 2.7 mequiv/g, cf. IEC (Nafion) = 0.9 mequiv/g.

Ionomeric coatings like Nafion have already been applied in Li metal batteries as an artificial SEI^56^ due to their high intrinsic ionic conductivity stemming from the sulfonyl group (−SO_3_^–^ H^+^). In this context, a high ion exchange capacity (IEC), i.e., a large ratio of sulfonyl groups per molecular weight, is desired. Therefore, Aquivion (IEC = 1.3 mequiv/g) and SPEEK (IEC = 2.7 mequiv/g) were chosen over Nafion (IEC = 0.9 mequiv/g) to enhance the ionic conductivity of the coating (Figure 1). It was attempted to exchange the protons from the sulfonyl group (−SO_3_^–^ H^+^) with Mg^2+^ cations by immersing the Aquivion powder in aqueous MgCl_2_ solution. However, such magnesiation results in cross-linking of ionomer chains (−SO_3_^–^, Mg^2+^, ^–^O_3_S−) and gelation, which precludes dissolution and the subsequent preparation of a coating solution. Therefore, Aquivion and SPEEK were used in their protonated form, and an in situ cation exchange is expected to take place during cell operation. In addition to the ionomeric approach, a coating based on cyclized PAN, which has already proven to work in Mg batteries,^40^ was prepared. In contrast to the previous study, no conductive carbon is necessary herein.

**Figure 2:**
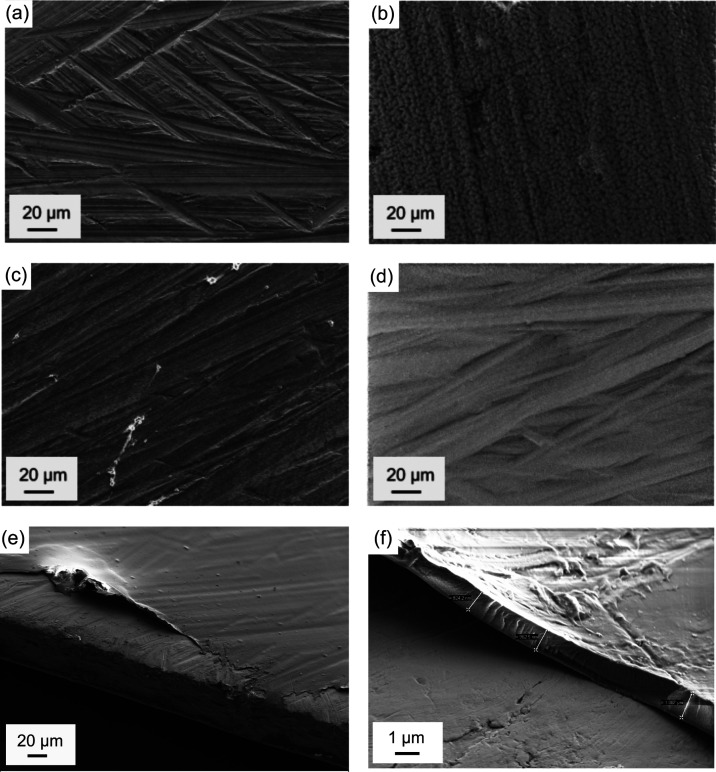
Surface morphology of (a) scraped Mg, (b) Mg-Aquivion/PVDF, (c) Mg-SPEEK/PVDF, and (d) Mg-PAN. (e) Poor edge coverage and (f) thickness approximation in the case of the PAN-coating (∼ 1 μm) on the Mg substrate (∼100 μm).

Spin-coating represents a simple technique to achieve thin and homogeneous coatings but requires the adjustment of several parameters. Besides the apparatus settings of spin speed and duration, the solution properties (viscosity, molarity, and polarity) in combination with the substrate properties (surface roughness and polarity) are crucial to the properties of the developed coating. With the above depicted experimental procedure, a uniform coverage of the Mg surface was achieved—see Figure 2a–d and Table S1. Note that the surface roughness was caused by thorough scraping to remove the native oxide layer. The coating thickness was determined to have a radial gradient with 1–5 μm at the anode rim and below 0.5 μm in the center (Figures S3 and S4) due to the coating solution being rotationally ejected. Furthermore, the rim could partially remain uncoated (Figure 2e,f). Such radial gradients can be overcome by other methods like dip- or spray-coating, which are furthermore scalable coating techniques—in contrast to spin-coating. However, spin-coating remains superior in achieving desired thicknesses below 1 μm (discussed below).

**Figure 3:**
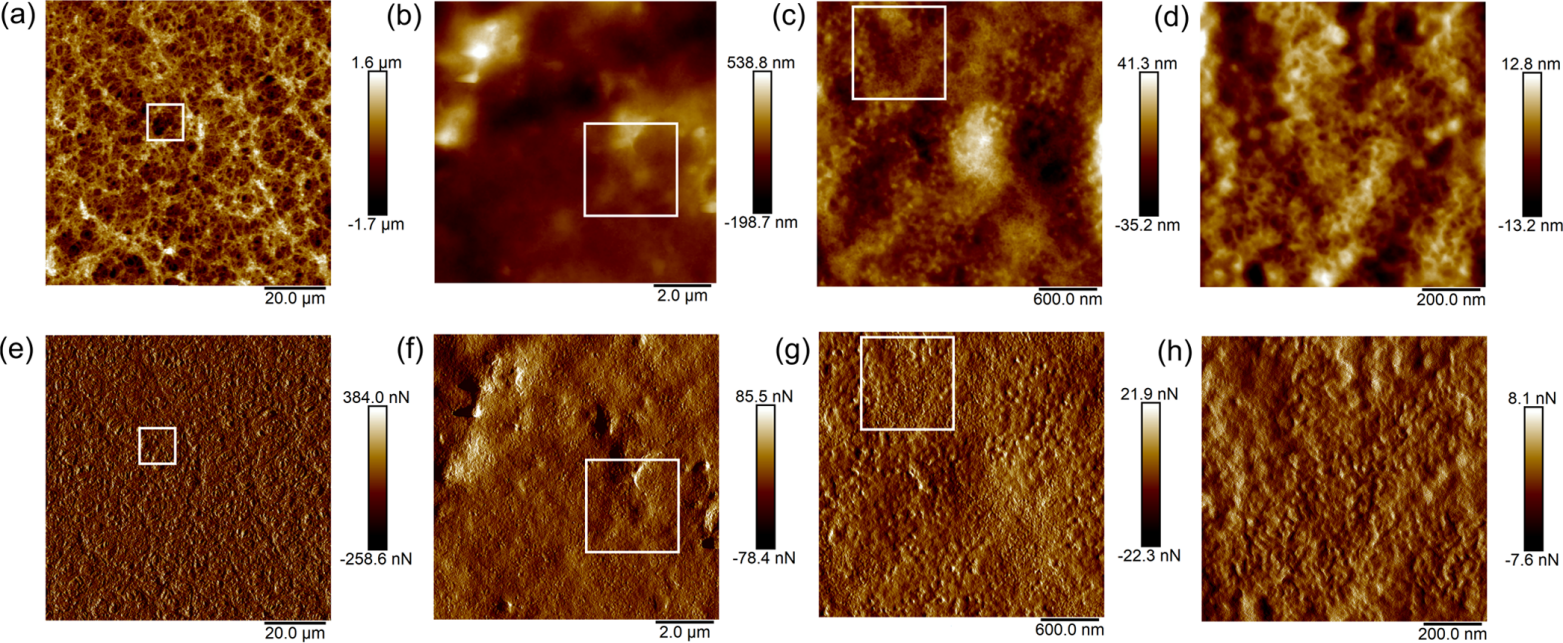
AFM images of Mg-Aquivion/PVDF (spin-coated) revealing the porous macro- (left) and microstructures (right) with the white frames indicating the zoomed areas. (a–d) Height sensor and (e–h) peak force error.

Additional insights into the coating morphology are gained via atomic force microscopy (AFM), which was used in previous studies to investigate ionomers in electrolysis^57^ and fuel cells.^58^ In the case of the Aquivion/PVDF coating, a rather rough surface with a sponge-like structure (Figure 3a–c) was observed (cf. cloud-like morphology in Figure 2b). At higher magnifications, a nanostructured morphology exhibiting pore sizes of approximately 10–50 nm becomes obvious (Figure 3d). Figure 3e–h depicts the complementary peak force error images which show the surface structures. In the case of lower thicknesses, solely micropores are present (Figure S5).

### 3.2 Polarization and EIS Measurement in Mg–Mg Cells

**Figure 4:**
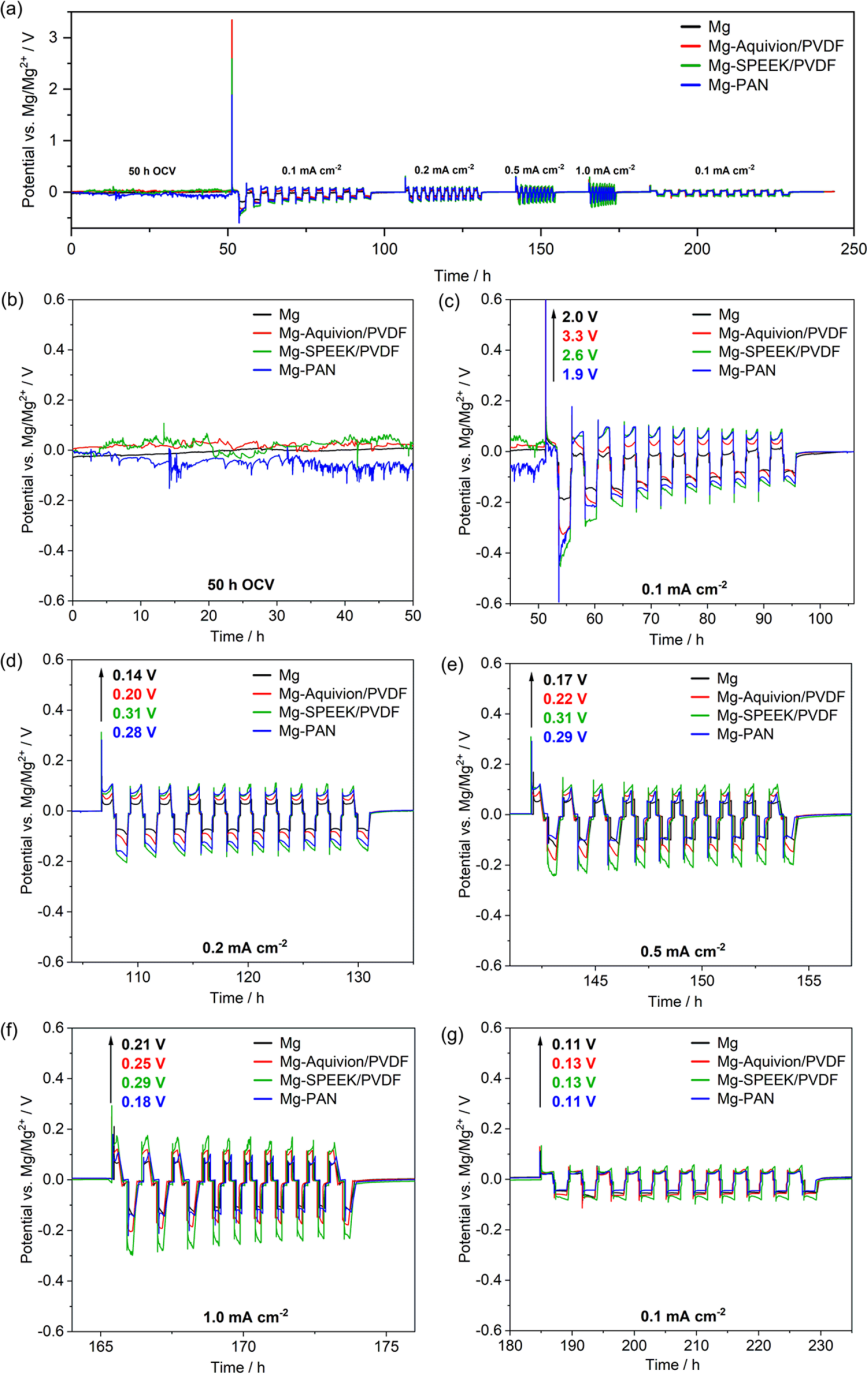
Potential profiles of symmetrical Mg–Mg cells with pristine and coated Mg electrodes (vs Mg RE). (a) Polarization at 0.1, 0.2, 0.5, 1.0, and final 0.1 mA cm^–2^ with an initial 50 and 10 h intermediate rest at OCV. Potential trend during (b) 50 h OCV and (c–g) polarization cycles at different current densities with evolving voltage spike and overpotential asymmetry during stripping and plating.

The coated Mg anodes were assembled in Mg–Mg cells to investigate the electrochemical properties of the artificial SEI (a-SEI) during polarization. To distinguish between stripping and plating, a Mg reference ring was utilized and a test procedure with different current densities and intermediate OCV periods was defined (Figure 4a). Electrochemical impedance spectra were collected in potentio- and pseudo-galvanostatic mode during OCV (each hour) and polarization (during cycles 1–3 and 10), respectively.

**Figure 5:**
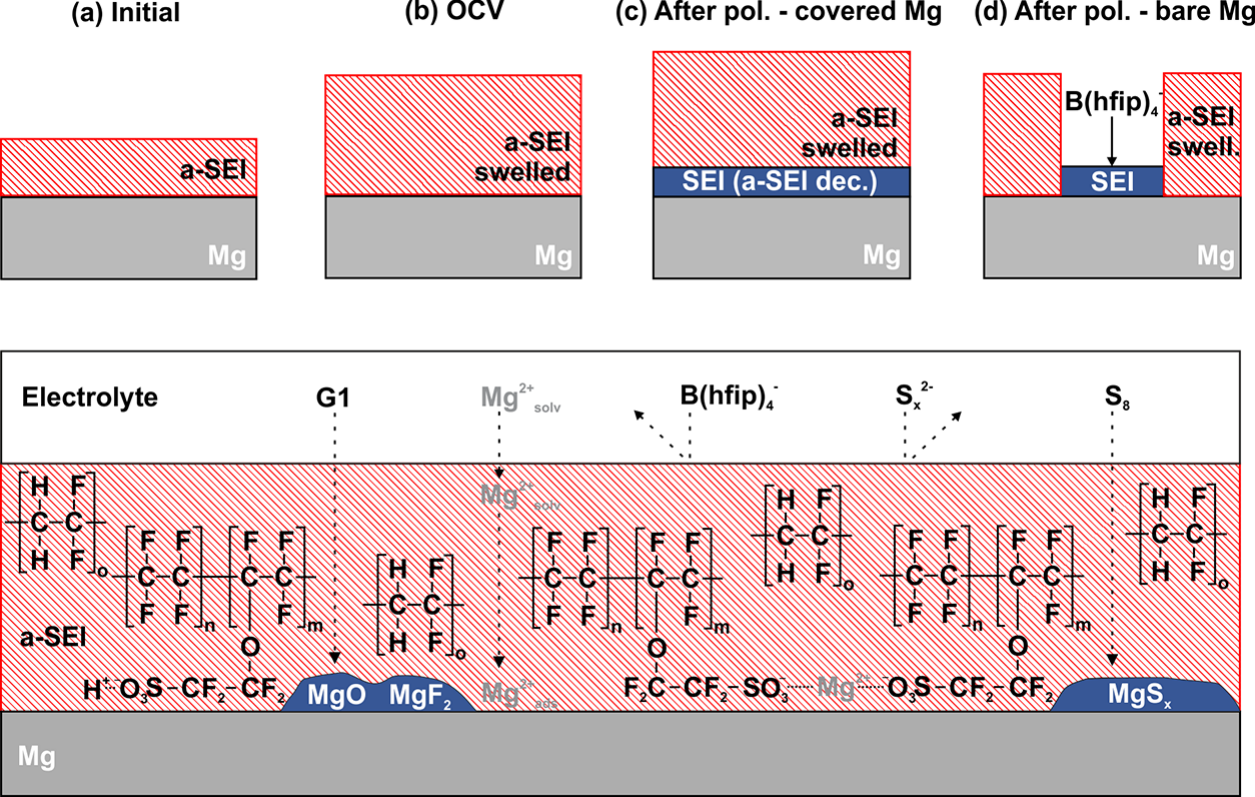
Schematic interface of a-SEI-coated Mg anodes: (a) initially, (b) during extended OCV in contact with electrolyte, (c) after polarization with a closed a-SEI layer and an in situ SEI formed underneath via polymer decomposition, and (d) coating failure due to morphology changes and in situ SEI formation via B(hfip)_4_^–^ decomposition beneath the a-SEI. Bottom: scheme of the Mg surface covered with an Aquivion/PVDF layer. In the case of an intact a-SEI, polysulfides and B(hfip)_4_^–^ anions are repelled. Nevertheless, an in situ SEI composed of MgO, MgF_2_, and MgS is likely via solvent^61^ and polymer decomposition.

The OCV profiles with significant deviations from the Mg/Mg^2+^ reference potential (Figure 4b) indicate that a uniform coating is present, which alters the anode potential. The potential fluctuations are probably originated in the swelling of the porous ionomer/polymer coating (cf. Figure 3) and only localized contact of bare magnesium with the electrolyte (Figure 5a,b). Despite showing a significantly smoother trend, the potential of the pristine Mg anode also deviates from zero, indicating the non-faradaic SEI formation on bare Mg in contact with the electrolyte at OCV. Note, that such a surface effect also concerns the reference electrode—despite non-current conditions. The topic of a reliable metal RE is critical^59,60^ but beyond the scope of this manuscript and will be addressed in a subsequent study.

The potential trends during stripping and plating are depicted in Figure 4c–g for the particular polarizations. In the initial ten cycles, a drift in equilibrium potential is observed, which is assigned to a conditioning of the Mg surface due to its reduction potential and reactivity toward species in direct contact (electrolyte or a-SEI). After ten cycles, the electrode potential, i.e., surface layer, appears to be stable, and the value after subsequent 10 h OCV was set as the equilibrium potential. Interestingly, potential fluctuations as observed in the initial OCV period do no further appear, indicating a current-induced surface modification, i.e., the in situ SEI formation composed of MgO and MgF_2_^3,4^ underneath the artificial SEI (Figure 5c) via decomposition of G1,^61^ PVDF,^4^ or Aquivion, while the anion diffusion and reduction at the Mg surface is mitigated (Figure 5, bottom).

**Figure 6:**
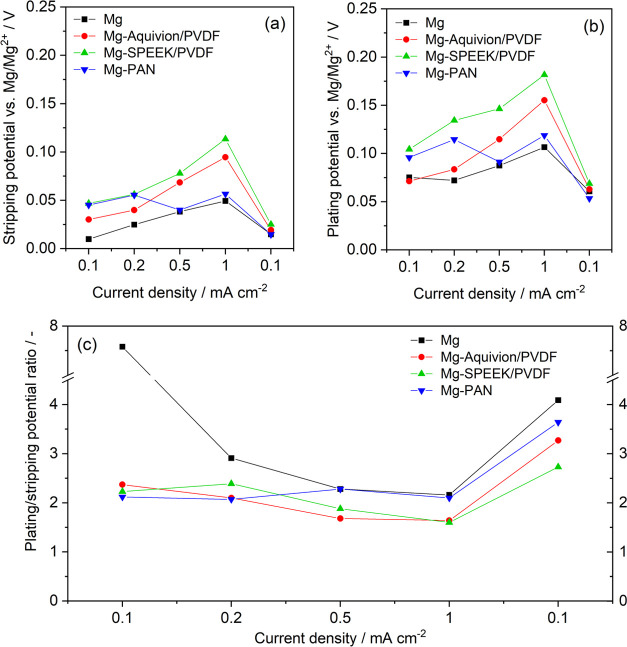
(a) Stripping and (b) plating potential during the final cycle of each polarization interval. (c) Current-dependent asymmetry in the plating and stripping potential for all investigated Mg anodes.

While the coated Mg anodes exhibit larger initial overpotentials compared to the pristine Mg, the differences become almost negligible in the final polarization cycles at 0.1 A cm^–2^ with the significantly decreased overall overpotential (Figure 6a,b). This gives hint to an increased surface area due to uneven Mg stripping/plating but also partial coating failure to result in in situ SEI formation via B(hfip)_4_^–^ decomposition (Figure 5d), facilitating the stripping/plating process. As expected, the polarization potential increases with current density, but interestingly, the ratio between stripping and plating also reveals a current dependency with increasing plating/stripping asymmetry, i.e., hindered Mg plating, at lower current densities (Figure 6c). This asymmetry was already observed in our previous simulation-backed study and is mainly originated in the high desolvation energy during Mg deposition.^62^ As the coated anodes exhibit the same asymmetry yet higher overpotentials, it is concluded that the coating does not affect the desolvation but the ion diffusion toward the Mg surface. A rather unexpected behavior was observed during potential relaxation: independent of a Mg coating, the potential after Mg stripping features a dip to approach the equilibrium from negative values—similar to the potential after plating (Figure S6). However, as the present Mg^2+^ concentration at the magnesium surface is high or low depending on stripping or plating (Figure S7), a different potential trend should result. Due to being reproducible, a measurement artifact is unlikely, but the actual origin remains unclear.

Additionally, a long-term polarization was performed for Mg and Mg-Aquivion/PVDF at 0.1 and 1.0 mA cm^–2^ (Figure S8). At both current densities, the ionomeric coating shows faster cell failure due to short circuits and generally higher overpotentials. However, the overpotential is constantly declining, while it is increasing in the case of the pristine Mg anode. The latter was previously observed in other studies and assigned to the partial decomposition of the salt and unstable SEI during prolonged cycling.^26,54^ The overpotentials match after 350 h at 0.1 mA cm^–2^, which again indicates the in situ formation of a similar ion-conductive SEI composed of decomposition products from the B(hfip)_4_^–^ anion beneath the artificial SEI (Figure 5d).

**Figure 7:**
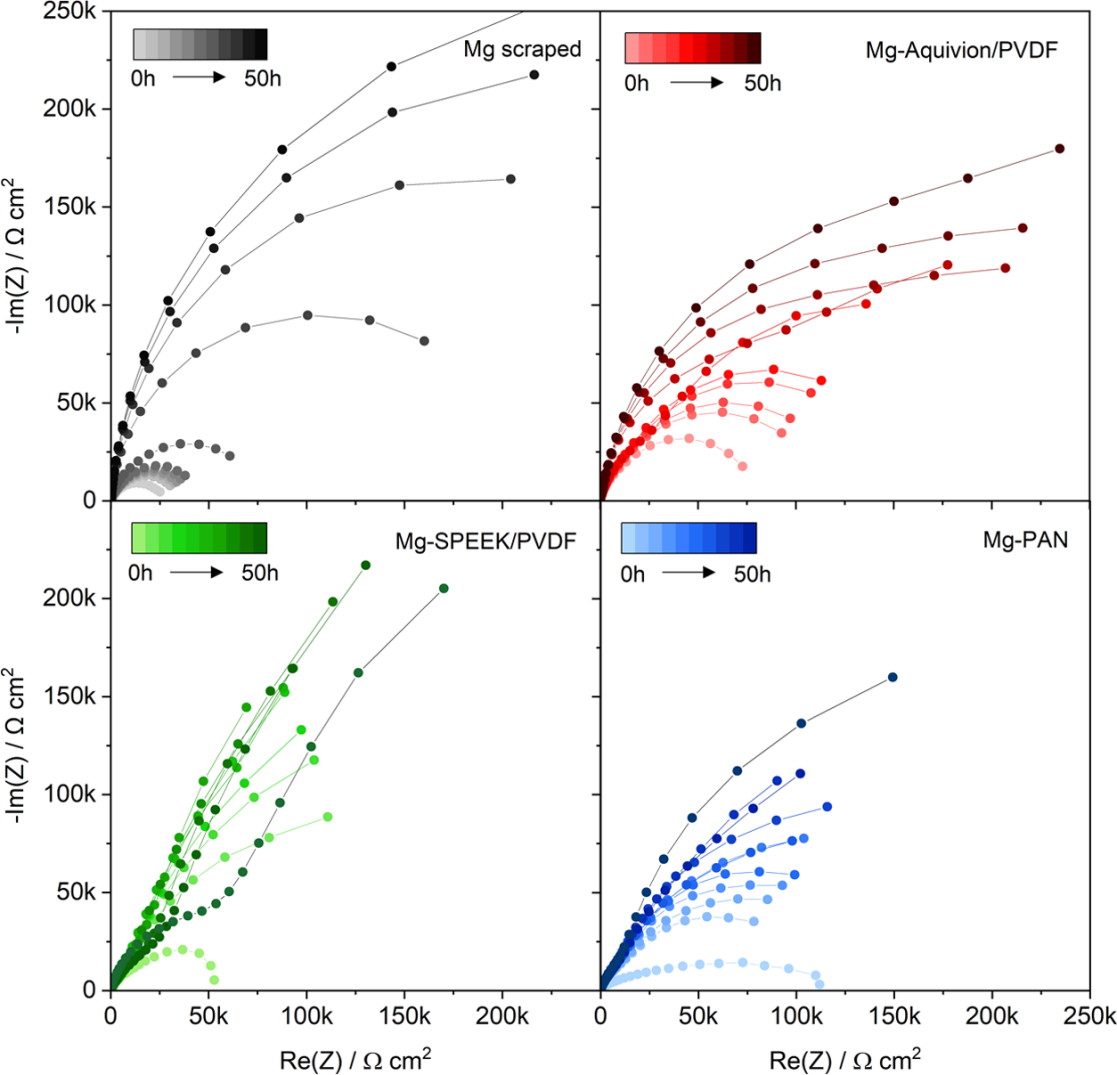
Nyquist plots of potentiostatic impedance spectra of pristine and coated Mg anodes during the initial 50 h OCV (cf. Figure 4b) applying a 5 mV amplitude in a frequency range of 300 kHz to 0.1 Hz.

To gain further insights, EIS measurements were performed during OCV and the polarization cycles. The Nyquist plots of the impedance spectra collected during the initial 50 h OCV of the pristine and coated Mg anodes are depicted in Figure 7. For all anodes, the cell impedance increases from 25 to 100 kΩ cm^2^ to several 100 kΩ cm^2^. This was already observed in previous studies about Mg metal^4,63^ and is assigned to the adsorption of electrolyte species at the Mg surface. While the trend in impedance is similar, the scraped Mg anode exhibits the lowest and largest cell impedance after cell assembly (0 h) and 50 h OCV, respectively. This indicates the impact of an a-SEI coating as it might induce a migration barrier but also mitigates the direct adsorption (and potential decomposition) of solvent molecules at the Mg surface.

**Figure 8:**
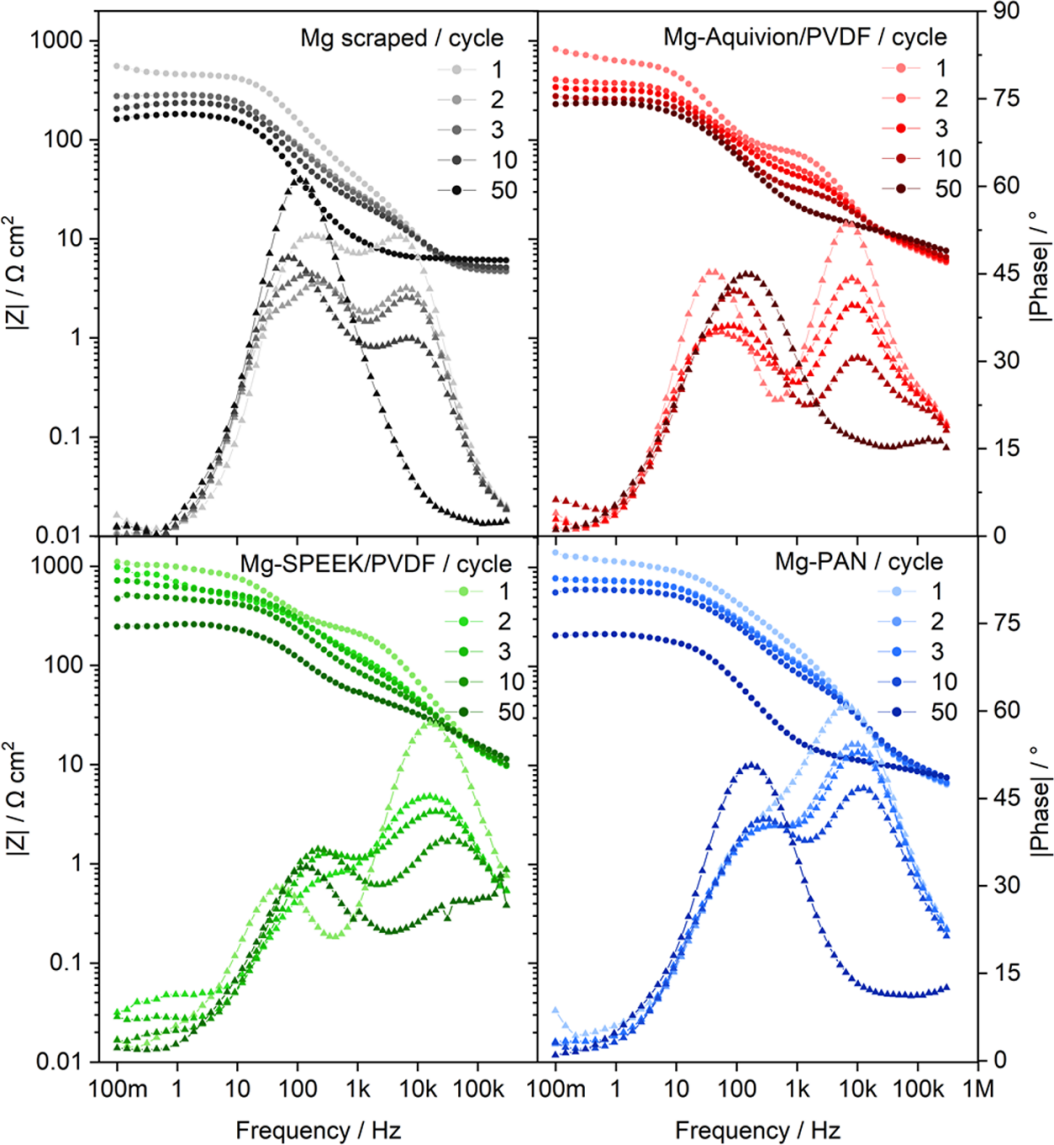
Bode plots of galvanostatic impedance spectra of pristine and coated Mg anodes during stripping at 0.1 mA cm^–2^ (polarization in Figure 4c,g) applying a 5 mV amplitude in a frequency range of 300 kHz to 0.1 Hz.

The Bode and Nyquist plots of the impedance spectra collected during the initial and final stripping cycles at 0.1 mA cm^–2^ for the pristine and coated Mg anodes are depicted in Figures 8 and S9, respectively. In this case, galvanostatic EIS mode (*I* = 0.1 mA cm^–2^, Δ*U* = 5 mV) was selected to avoid the high-Ohmic “adsorption layer” and reveal the practical impedance values during cell operation. In general, the pristine Mg anode exhibits the lowest impedance, which is in direct correlation with the lowest overpotentials. In contrast to the bare magnesium, the coated anodes feature an additional impedance contribution above 50 kHz, which is assigned to the porous polymeric network on the Mg surface.

Apart from that, two main processes can be identified with the process in the high-frequency region vanishing during polarization—again most probably due to an increase in anode surface area and a consequential decrease of the corresponding impedance. Note that the geometrical electrode area (2.545 cm^2^) was chosen for the initial normalization. As a consequence of the larger surface area, the overall impedance declines with proceeding polarization for all anodes. When comparing the impedance spectra 10 h after cell assembly and polarization (Figure S10), the high-Ohmic adsorption layer becomes less dominant after the first polarization cycle—partially due to an increased surface area but probably also due to less adsorption on the in situ formed SEI on Mg. Further insights by a process assignment and detailed EIS analysis will be given in a subsequent study.

### 3.3 Galvanostatic Cycling of Mg–S Cells

**Figure 9:**
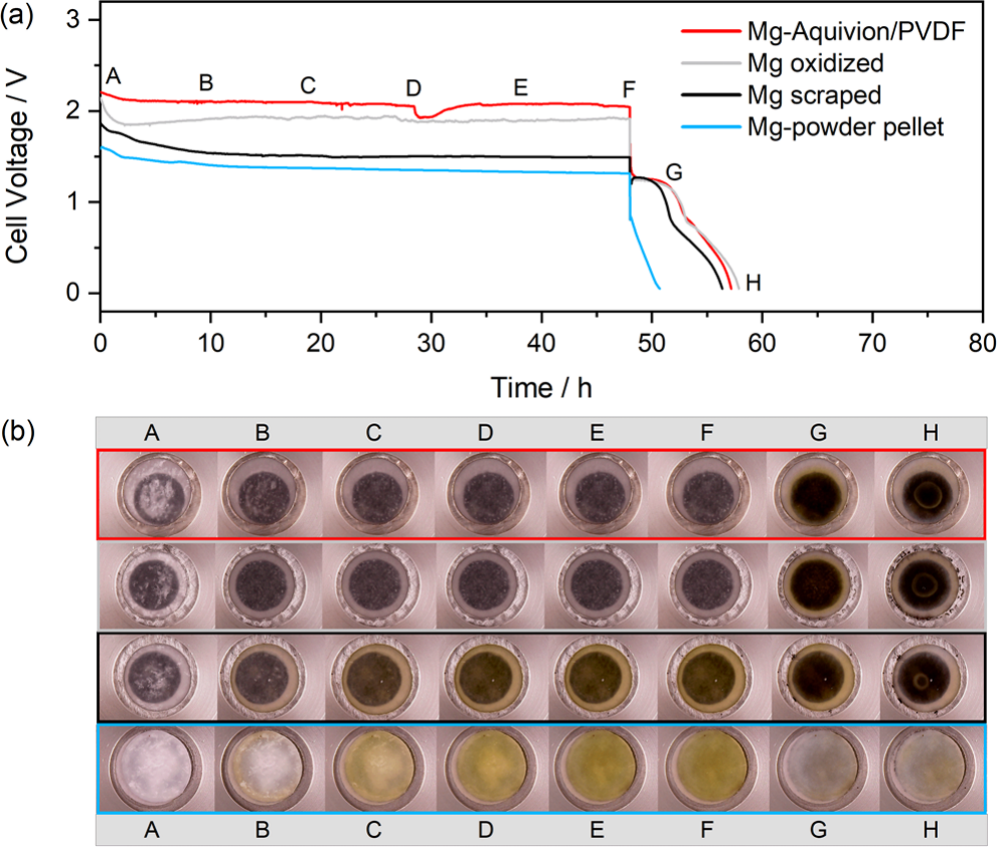
(a) 48 h OCV with subsequent discharge at *C*/20 and (b) corresponding optical cell images collected during operation. Different Mg anodes in an otherwise identical optical cell setup were applied (50:40:10 wt % S/KB/CMC-SBR cathode, GF/C separator, 0.2 M Mg[B(hfip)_4_]_2_/G1 electrolyte). In the case of the Mg pellet, an additional separator was used.^2^

Indeed, the use of an organic artificial SEI solely rated by their performance in the Mg–Mg cells is not beneficial. However, the performance in Mg–S cells is equally important, as the major motivation for an artificial SEI in this study is the hindrance of the direct contact of dissolved sulfur species in the electrolyte with the Mg surface to diminish the polysulfide shuttle and ongoing parasitic reactions. This was indeed confirmed by operando imaging during the 48 h OCV and subsequent cycling of Mg–S cells applying different Mg anodes (Figure 9). The cells comprising a scraped Mg foil or a pellet pressed from Mg powder feature a fast potential decline accompanied with yellowish coloration of the electrolyte-wetted separator due to the non-faradaic formation of polysulfides at the bare Mg surface. This is the consequence of fast sulfur dissolution and diffusion to the anode without significant sulfur retention at the cathode.^2^ In contrast, the cell with a Mg-Aquivion/PVDF anode does not exhibit a yellow coloration, i.e., no polysulfides are present in the electrolyte. However, as an ionomeric coating only repels anions, S_8_ should be capable to diffuse through the a-SEI to be reduced at the Mg surface (Figure 5, bottom). Yet, the formation of polysulfides is inhibited and the shuttle effect is suppressed due to the repelling charge of the sulfonated groups. This is evident by the still rather high cell voltage after 48 h OCV. Interestingly, an oxidized Mg anode surface shows a similar behavior, i.e., the contact of sulfur with bare Mg is hindered by the MgO layer.

**Figure 10:**
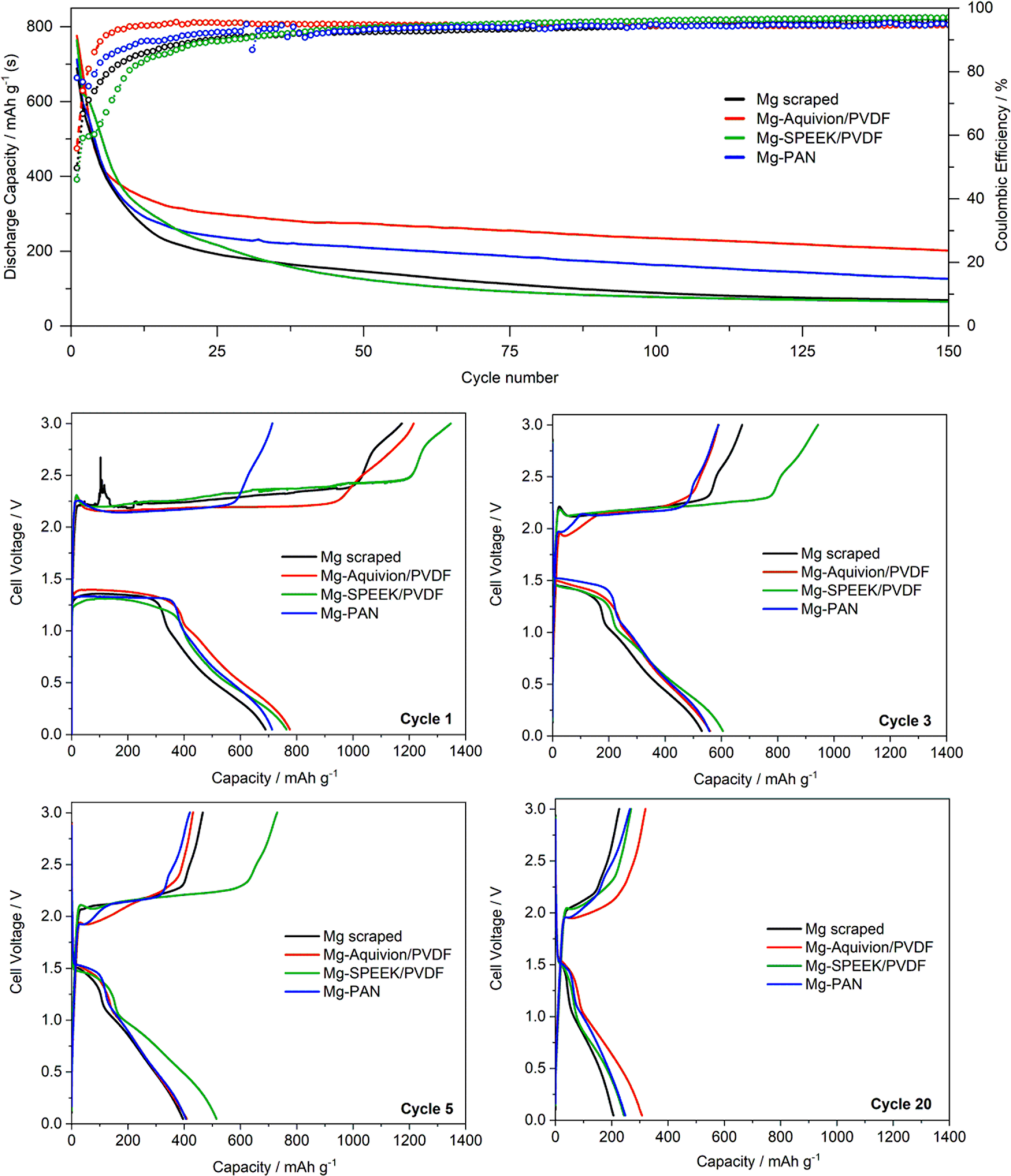
Comparison of pristine and coated Mg anodes in Mg–S cells cycled at *C*/10.

The discharge capacity and Coulombic efficiency during prolonged cycling of Mg–S cells comprising pristine and coated Mg anodes are depicted in Figure 10. All cells feature a steep initial capacity decay and low Coulombic efficiency in the initial cycles typical of metal-sulfur batteries associated with the abovementioned basic cathode approach, i.e., partial active material loss in the electrolyte or by the formation of magnesium sulfide. After approximately 10 cycles, this parasitic reaction is diminished and the Coulombic efficiency as well as the discharge capacity stabilizes—yet the latter still constantly declines.

Herein, the cells with Mg-PAN and Mg-Aquivion/PVDF anodes outperform the uncoated anode with the latter exhibiting a more than doubled capacity gain after 150 cycles (201–69 mAh/g). This is again due to the fact that the reductive magnesium surface is (mostly) covered by the polymer film hindering the direct contact with polysulfides and their undesired reduction reaction at the anode surface. This is already obvious in the first cycle (Figure 10, cycle 1), in which all cells with coated anodes feature a longer first discharge plateau—consequently less previous self-discharge. During subsequent charge, the potential trend of cells with coated anodes is smoother with a lower overpotential in the case of Mg-Aquivion/PVDF and Mg-PAN pointing to a diminished electrolyte salt decomposition and polysulfide shuttle due to the no/less bare magnesium surface (Figure 5). This is further backed by the fact that despite a shorter charge plateau, subsequently more discharge capacity can be gained—indicating less parasitic charge consumption (Figure 10, cycle 3). Consequently, the initial Coulombic efficiency is significantly increased compared to a bare Mg anode. In subsequent cycles, an additional plateau with a lower overpotential at the beginning of charge is present, which indicates facilitated Mg^2+^ desolvation or enhanced Mg plating underneath the artificial SEI. The Mg-SPEEK/PVDF shows the opposite behavior: it provides the largest capacity gain in the initial cycles but at the lowest Coulombic efficiency and increased charge (and discharge) overpotentials. As the properties of Aquivion and SPEEK should be comparable according to their related structure, this either points to an insufficient coating homogeneity—despite identical solution concentration and processing, or reflects a stronger crosslinking, i.e. reduced ion mobility due to a higher IEC (discussed below).

Further insights into the surface morphology and composition are gained with post mortem SEM and EDX analysis (Figure S11 and Table S1). It was found that the Mg stripping/plating reactions preferentially occur at the anode rim/edges (Figure S11)—probably due to the higher local current density in those areas. A roughened and porous anode surface with macroscopic holes remains indicating that a certain amount of magnesium is lost/inactive in the separator. The reaction zones exhibit black coloration due to the simultaneous reduction of the electrolyte and sulfur to MgF_2_, MgO, MgS, and/or MgSO_4_ shown in our previous XPS study.^4^ The anodes with an artificial SEI feature a rather similar morphology with the edges still being prone to react with the electrolyte and sulfur species. Such an edge effect might be overcome with other coating techniques such as dip-coating^64^ and overall becomes less crucial in larger cells.

**Figure 11:**
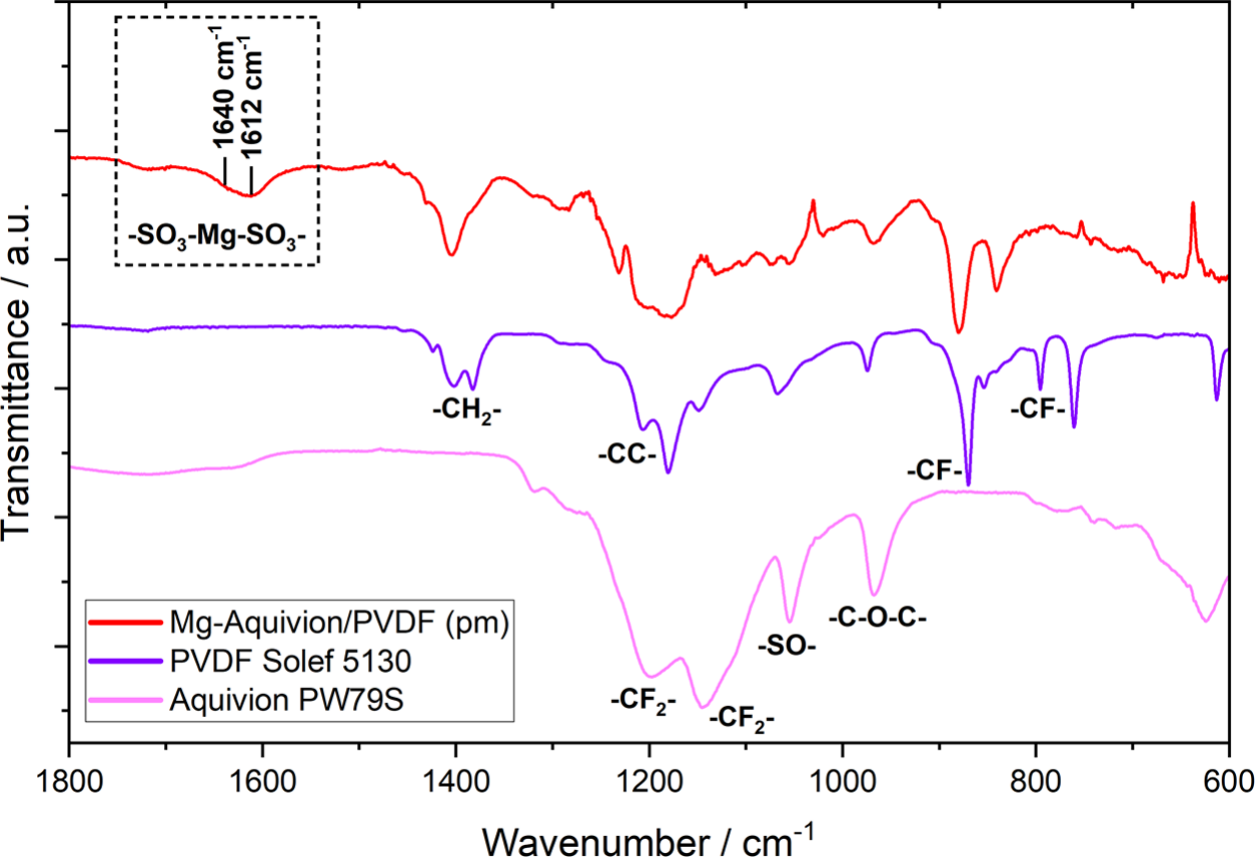
Fourier transform infrared (FTIR) spectra of a Mg-Aquivion/PVDF anode (Mg–S cell, post mortem) in comparison to PVDF and Aquivion powder. Partial in situ cation exchange and consequent cross-linking of the SO_3_^–^ groups are observed.

Due to Mg-Aquivion/PVDF showing the most promising results, it was attempted to gain further insights by additional measurements. As mentioned above, an ex situ cation exchange results in cross-linking and insolubility; thus, it is supposed to partially occur in situ during cycling. Indeed, this was confirmed via IR post mortem analysis of Aquivion/PVDF-coated anodes (Figure 11), wherein the vibrational bands at approximately 1612 and 1640 cm^–1^ are assigned to Mg^2+^ substituting H^+^ in the SO_3_^–^ group. Similar was observed for a Li^+^ exchange at 1630 cm^–1^.^65,66^ In this regard, the cross-linking of Aquivion chains is beneficial to enhance the mechanical strength of the surface layer and its chemical immobilization (Figure 5). In contrast, the ion mobility in the ionomer might be in fact hampered due to the strong cross-linking. This is supposed to be the origin of the poor performance of the Mg-SPEEK/PVDF anode as the cross-linking therein is even more pronounced (Figure S12).

**Figure 12:**
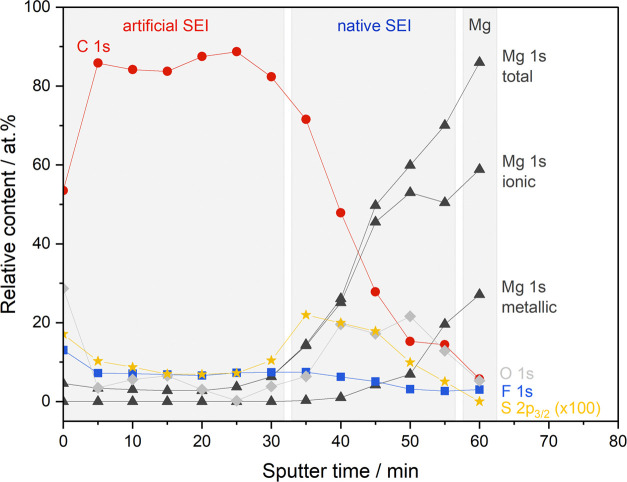
XPS depth profile of a Mg-Aquivion/PVDF anode cycled at *C*/10 for 150 cycles in a Mg–S cell with a 0.2 M Mg[B(hfip)_4_]_2_/G1 electrolyte.

To reveal the distinct composition of the anode interphase, XPS depth profiling of a post mortem Mg-Aquivion/PVDF anode cycled for 150 cycles at *C*/10 in a Mg–S cell was carried out. The nonwashed anode was transferred to the XPS chamber via an inert gas module, and a position in the electrode center was selected for investigation. The trend of the relative content of C, O, F, Mg, and S is displayed in Figure 12 with the corresponding spectra being listed in Figure S13. The first data point each (0 s) corresponds to the pristine sample with remaining surface contaminants and an obvious local charging, shifting all signals by a few eV (see Figure S13).

A rather constant ratio of C, O, F, Mg, and S was detected at low sputter times, wherein carbon is dominating the composition of the outer surface layer (>80 at %) with an oxygen and fluorine content of 5–10 at %. As this large C amount cannot solely be originated in electrolyte salt residues and the G1 solvent molecules are volatile, this reflects the polymeric coating. However, neither the C 1s nor the F 1s spectra (Figure S13) point to fluorine bound to a polymer backbone (C–F); thus, it is supposed that the coating layer is altered/decomposed during cycling. It further cannot be excluded that the susceptibility of the C–F bond to ion beam etching and X-ray radiation leads to a significant loss during the extended depth profiling.^67^ A rather low sulfur content of 0.1–0.2 at % was observed, which might stem from the SO_3_^–^ ionomeric groups and confirms the coating to effectively mitigate the diffusion of polysulfides. The promising approach of an ion-selective artificial SEI is further backed by the fact of no boron being detected. Interestingly, the Mg 1s spectra indicate an ionic Mg content of 3–5 at % within the coating layer—most probably as cross-linked −SO_3_–Mg–SO_3_– groups or decomposition products MgF_2_ and MgO. After 30 min of sputtering, the Mg content increases with the surface composition being dominated by inorganic Mg compounds, i.e., the in situ formed SEI, which mainly consists of MgO and MgF_2_. Likewise to the oxygen amount, an increased content of sulfur was observed (at 35–45 min) with a corresponding transfer from polysulfide to monosulfide, visible by means of a shift in peak binding energy^4,68^ (Figure S13), indicating MgS being formed in close contact to the metallic Mg. Yet, MgS was present in larger quantities in our previous study with pristine Mg anodes.^4^ At higher sputter times, metallic Mg was observed underneath the in situ SEI layer.

**Figure 13:**
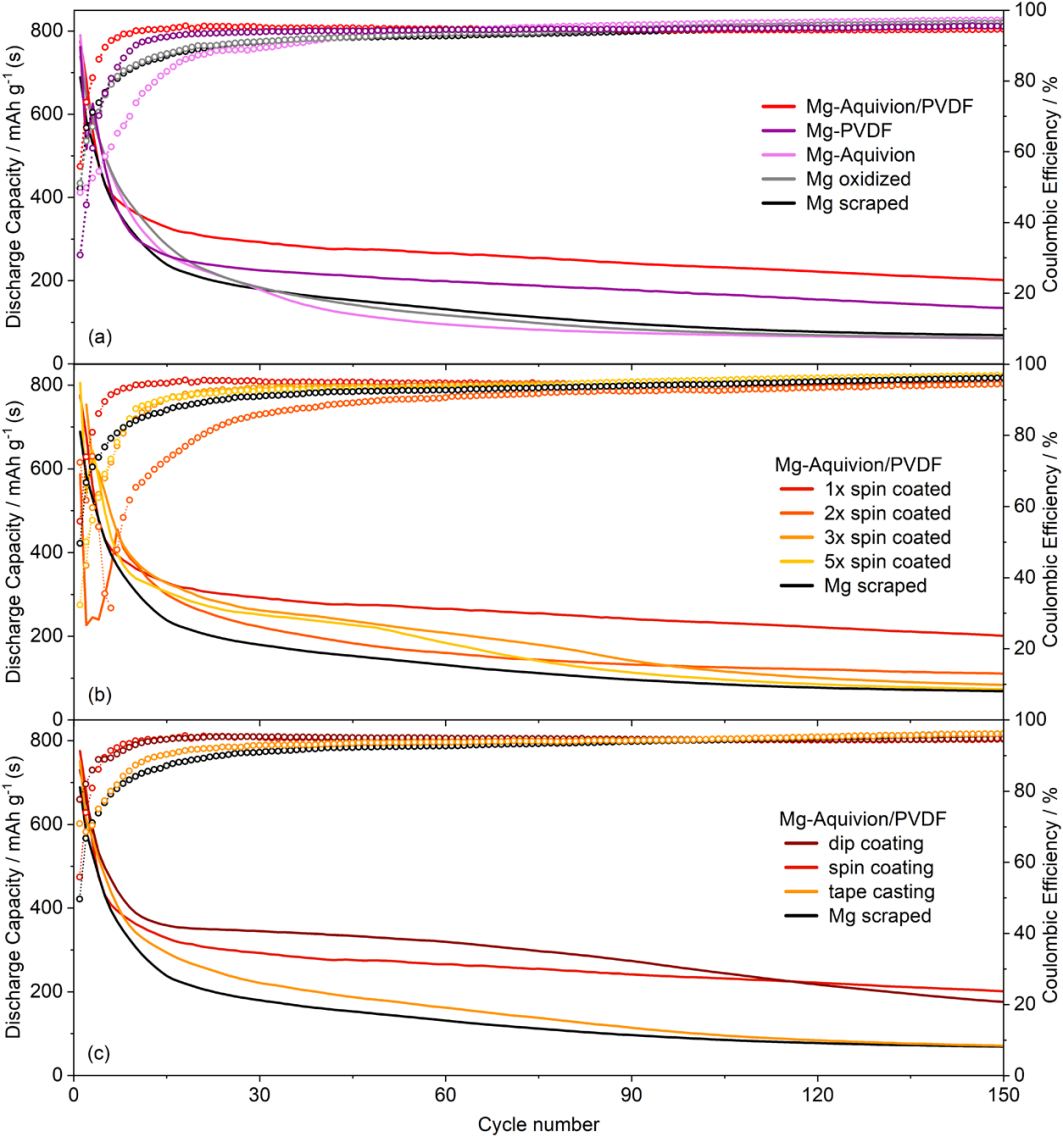
Discharge capacity and Coulombic efficiency of Mg–S cells cycled at *C*/10. Influence of (a) Mg foil oxidation and PVDF binder, (b) coating thickness and homogeneity, and (c) different coating techniques.

As previously observed by Luo et al. for Nafion in Li–S batteries,^56^ PVDF is a crucial component to ensure the mechanical stability of protective coatings and hinder the ionomer from dissolving into the ether-based electrolyte. A similar trend was found herein (Figure 13a) as Aquivion and Nafion hardly differ in the chemical structure and therefore feature similar properties. The dissolution occurs instantly with no chance for in situ cross-linking of Aquivion chains to fix the ionomer at the surface resulting in a strong capacity fade compared to the Mg-Aquivion/PVDF anode during cycling. With the bare magnesium surface being in contact with electrolyte/sulfur species, the cycling trend becomes similar to the pristine anode (Figure 13a).

Interestingly, the drying conditions also influence the homogeneity of the coating as fast drying, i.e., applying vacuum instantly after coating results in a transparent coating, while after slow drying under ambient conditions, white areas are observed. These exhibits either a rim (spin-coating), line (dip-coating), or dot form (tape casting). It is assumed that during solvent evaporation, the polymer chains are drawn into the remaining solvent by capillary forces to agglomerate there.^69^ As a consequence, pinholes or even larger areas of uncoated Mg are present causing an accelerated capacity decay.

Interestingly, the Mg foil scraped but subsequently oxidized for 24 h under an ambient atmosphere (Table S1) features a similar capacity trend than the Mg foil scraped under an Ar atmosphere in the glovebox. Moreover, the oxidized Mg surface is even beneficial as the initial capacity gain is in fact slightly larger (Figure 13a) and the charge potential is smoother (Figure S14) pointing to a mitigated reduction of the electrolyte and sulfur species at the bare magnesium metal (cf. Figure 9). According to previous studies, the oxide layer on Mg metal is only about 1.5 nm thick^70^ and rather stable, preventing further oxidation—similar to aluminum metal. Thus, Mg metal is well suited as the anode material in terms of processability as its reaction with humidity and oxygen is not detrimental and handling as well as coating under ambient conditions are feasible.

Figure 13b depicts the importance of surface layer homogeneity and thickness. While it is more likely to achieve a full anode coverage with multiple coating layers, it also raises the probability of cracks, sudden coating failures, and localized Mg stripping/plating. This is observed with anodes coated three or five times after approximately 80 and 50 cycles, respectively.

To investigate alternative coating techniques with industrial relevance, dip-coating, and tape casting were applied (Figure 13c), which are not restricted in substrate size and therefore scalable (Figure S15)—in contrast to spin-coating. Moreover, in the case of dip-coating,^64^ the electrode rims might be sufficiently coated, which diminishes the previously found edge effect (Figure S11). Thus, in comparison to the cell comprising the one-time spin-coated anode, a flat charge plateau (Figure S16), a similar Coulombic efficiency, and an even higher discharge capacity was achieved via dip-coating. However, after approximately 60 cycles, stronger capacity fading occurs, which is again attributed to a coating failure. The Aquivion/PVDF layer prepared via tape casting exhibits a larger thickness of approximately 1−2 μm and shows inferior cell performance. Careful adjustment of preparation parameters like doctor blade slit width along with polymer solution viscosity and concentration might improve the coating quality and electrochemical performance—yet this is out of the scope of this study. In general, dip-coating is favored as it is also suited to coat rough foils and three-dimensional anodes. Yet for instance, spin-coating is regarded superior in achieving homogeneous and, moreover, reproducible coatings.

## 4 Conclusions

In this study, two ionomers (Aquivion and SPEEK) were investigated in addition to a polymeric approach (PAN) to realize an organic artificial SEI (a-SEI) on Mg metal foil. In comparison to bare Mg, all coated anodes exhibit higher overpotentials in Mg–Mg cells, which indicates the in situ formed SEI to feature higher ion conductivity. Contrarily, in Mg–S cells, the Coulombic efficiency in the initial cycles and the discharge capacity during cycling are significantly increased applying coated Mg anodes. Furthermore, operando imaging during extended OCV indicates no separator coloration, hence less/no self-discharge. This confirms the a-SEI to be anion-repelling as the direct contact of polysulfides—and the electrolyte salt to form an in situ SEI—is mitigated. Consequently, Mg-Aquivion/PVDF anodes provide a more than doubled discharge capacity after 150 cycles compared to a pristine Mg anode (201–69 mAh/g). Furthermore, the in situ cross-linking of Aquivion chains—confirmed via post mortem IR analysis—is beneficial to enhance the mechanical stability, yet probably at the expense of ion mobility. Nevertheless, PVDF represents a crucial component to ensure the chemical stability of the a-SEI and hinder the ionomer from dissolution.

Remarkably, it was possible to prepare the coatings under ambient conditions, and an argon atmosphere was only necessary for cell assembly. Attention has to be paid regarding a uniform surface coverage to prevent bare Mg getting in contact with the electrolyte and sulfur species. Therein, spin-coating represents a suitable technique to realize thin coatings but is restricted to small electrodes, causes radial gradients, and exhibits poor/no edge coverage. Therefore, scalable techniques like tape casting and dip- and spray-coating should be applied in future. However, it has to be noted that bare Mg might be present in either case due to severe surface reactions during cell operation—causing macroscopic holes and local coating failure. In that context, a polymeric/ionomeric coating might be at least superior to an inorganic coating to withstand such anode morphology changes longer. Overall, an organic artificial SEI appears to be a promising component to facilitate the electrode preparation, counteract ongoing reduction at the anode surface, and enhance the performance of Mg–S batteries.

## Supporting Information Available

Spin-coating procedure; cell setup; electrochemical characterization results; AFM and SEM images; IR, EDX and XPS analysis to depict the artificial SEI morphology/thickness and chemical composition (PDF)
