# Supplementary material for: Magnesium Anode Protection by an Organic Artificial Solid Electrolyte Interphase for Magnesium-Sulfur Batteries
Source: ACS Appl Mater Interfaces. 2023 Jun 30;15(27):33013–27. doi: 10.1021/acsami.3c07223 (PMC10347424; doi:10.1021/acsami.3c07223)
Supplement: Supplementary file 1 — am3c07223_si_001.pdf [file am3c07223_si_001.pdf]

Supporting Information

Magnesium Anode Protection by An Organic Artificial Solid Electrolyte Interphase for Magnesium-Sulfur Batteries

Joachim Häcker,*[,a] Tobias Rommel,[a] Pia Lange,[a,b]
Zhirong Zhao-Karger,[c] Tobias Morawietz,[a,d] Indro Biswas,[a] Norbert Wagner,[a]
Maryam Nojabaee[a] and K. Andreas Friedrich[a,e]

* Corresponding Author
e-mail: joachim.haecker@dlr.de

[a] Institute of Engineering Thermodynamics, German Aerospace Center (DLR), Pfaffenwaldring 38-40, 70569 Stuttgart, Germany

[b] Institute of Inorganic Chemistry, University of Stuttgart, Pfaffenwaldring 55, 70569 Stuttgart, Germany

[c] Helmholtz Institute Ulm (HIU) Electrochemical Energy Storage, Helmholtzstrasse 11, 89081 Ulm, Germany

[d] Faculty of Science, Energy and Building Services, Esslingen University of Applied Sciences, Kanalstraße 33, 73728 Esslingen am Neckar, Germany

[e] Institute of Building Energetics, Thermal Engineering and Energy Storage (IGTE), University of Stuttgart, Pfaffenwaldring 6, 70569 Stuttgart, Germany

Spin coating procedure

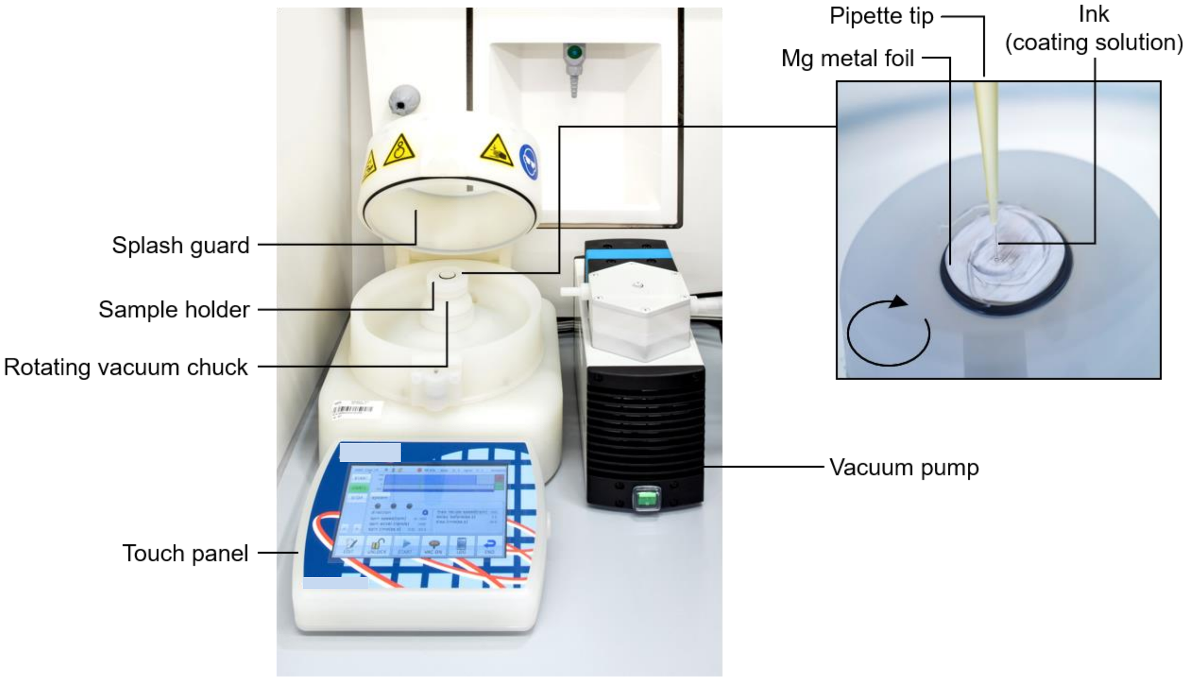

Figure S1: Preparation of ionomer coatings on magnesium electrodes (18 mm diameter) via dynamic spin coating applying a SPIN 150i spin coater (SPS). 150 µl polymer/ionomer solution and a spin speed of 1000 rpm was used.

Cell setup and EIS measurements

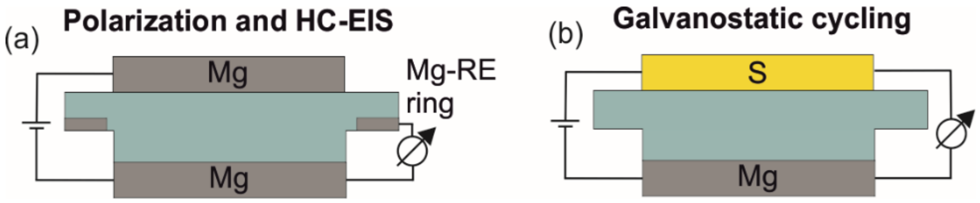

Figure S2: (a) Setup of the Mg-Mg cell (ECC PAT-Core, El-Cell) with Mg foil ring as reference electrode, two Mg anodes (pristine/coated), two separator sheets (Whatman GF/C) and 200 µl 0.2 M $Mg[B(hfip)_4]_2$/G1 electrolyte. The EIS measurements were performed vs. Mg-RE in potentio- or pseudo-galvanostatic mode with 5 mV amplitude. (b) Mg-S cell with identical setup – apart from the Mg-RE.

Estimation of coating thickness

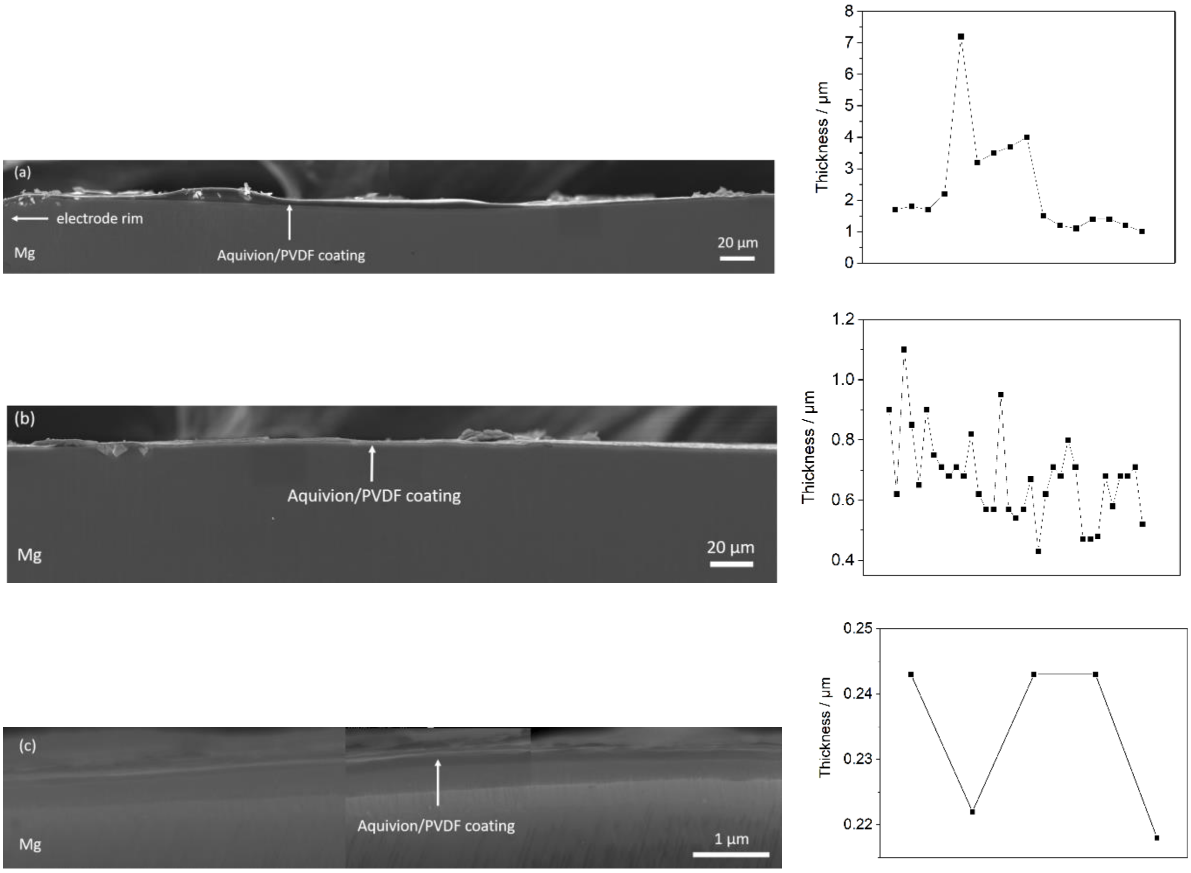

Figure S3: Cross-section images of ion-cutted Mg-Aquivion/PVDF electrodes reveal radial gradient in coating layer thickness from 1-5 µm at the electrode rim (a,b) to < 250 nm in the electrode center (c).

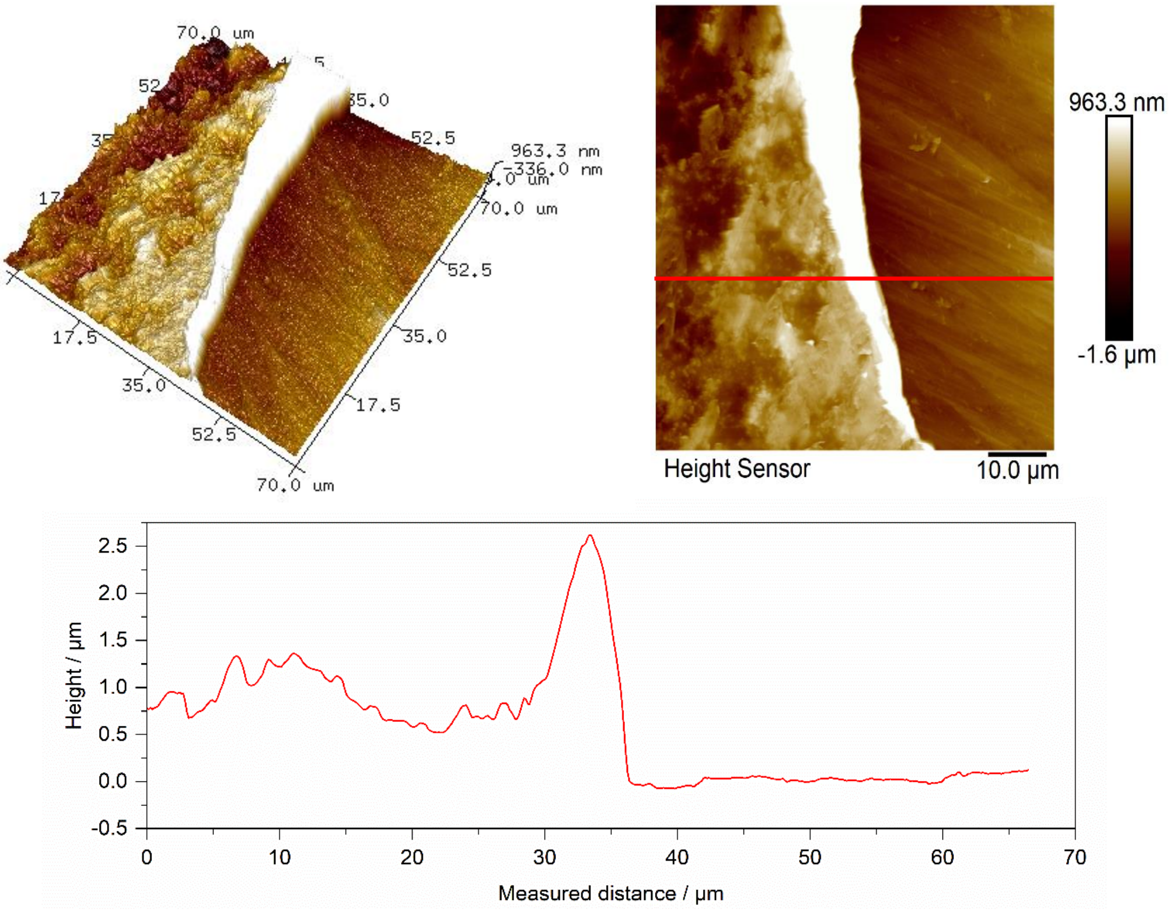

Figure S4: AFM image revealing the topography of Mg-Aquivion/PVDF (spin-coated) with the height profile data extracted along the red line.

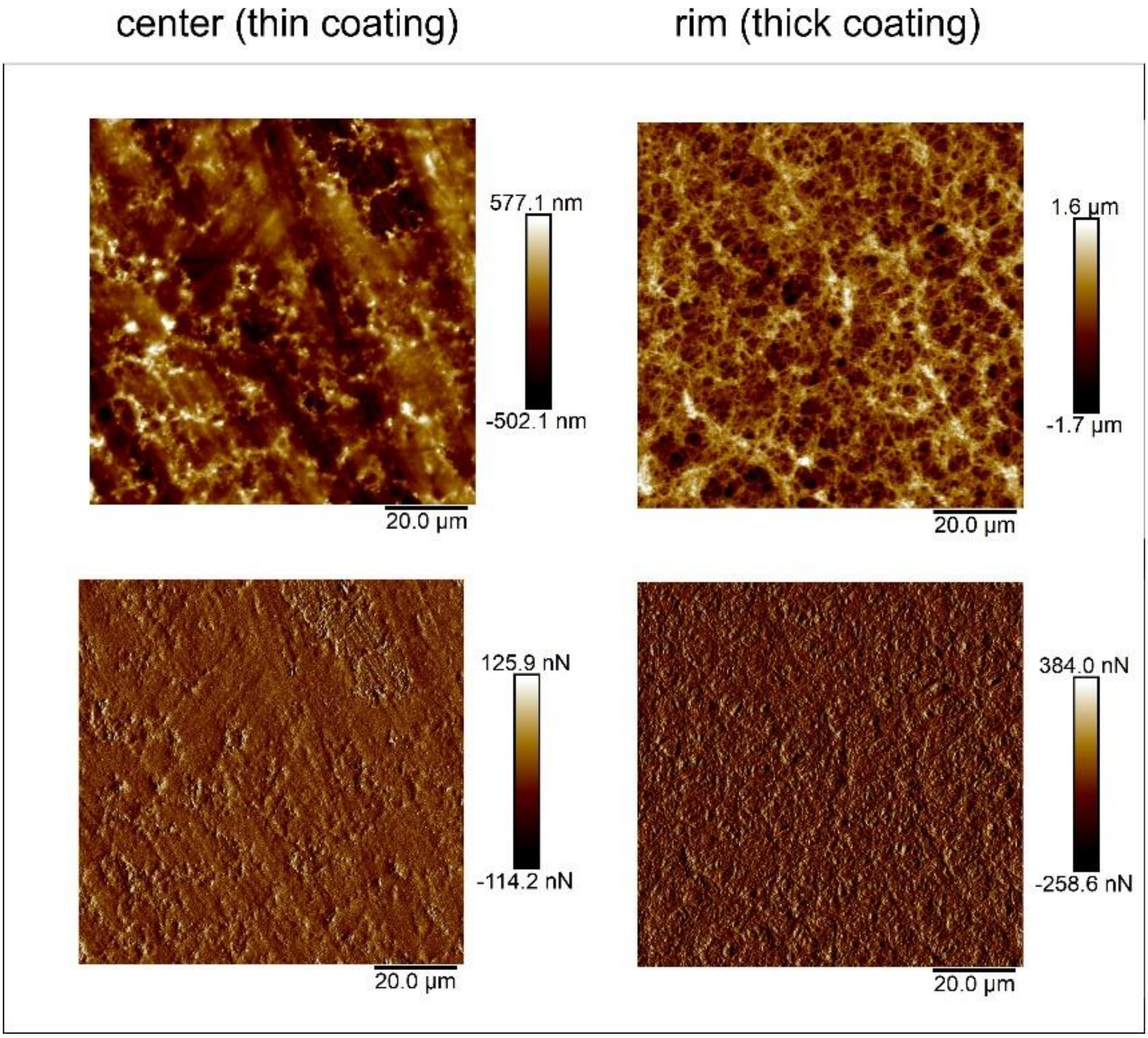

Figure S5: AFM images of Mg-Aquivion/PVDF (spin-coated). While the coating in the electrode center is rather thin and only exhibits microporosity, the thicker areas (visually white) at the electrode rim additionally feature a sponge-like macroporosity.

Polarization of pristine and coated Mg electrodes in Mg-Mg cells

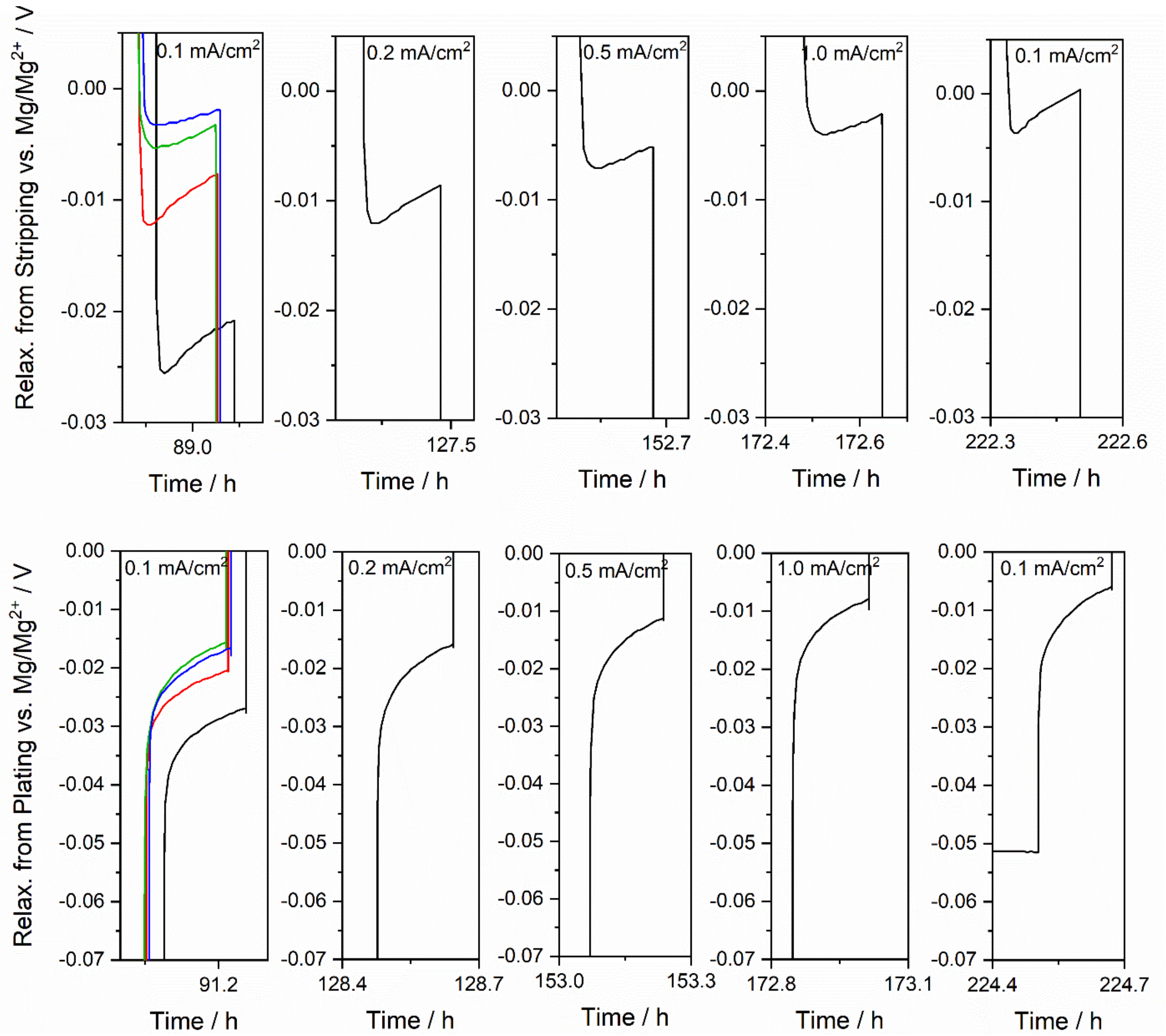

Figure S6: Potential relaxation from stripping (top) and plating (bottom) during polarization cycles (cycle 9) at different current densities of a Mg-Mg cell with pristine Mg anodes vs. Mg RE.

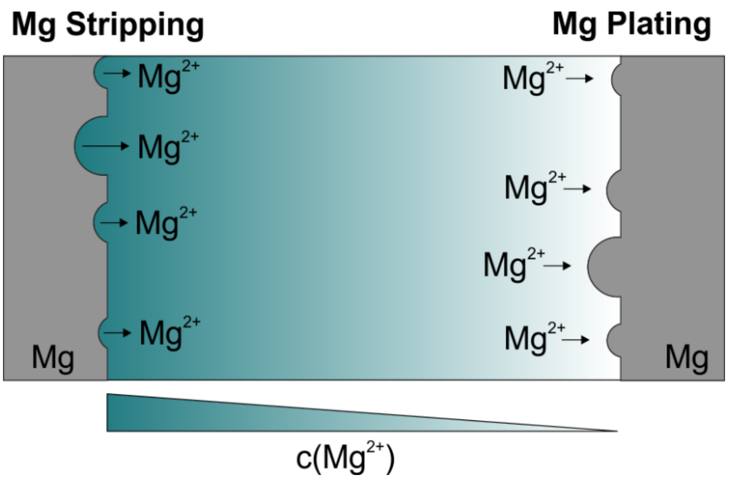

Figure S7: Schematic Mg-ion concentration gradient during stripping and plating.

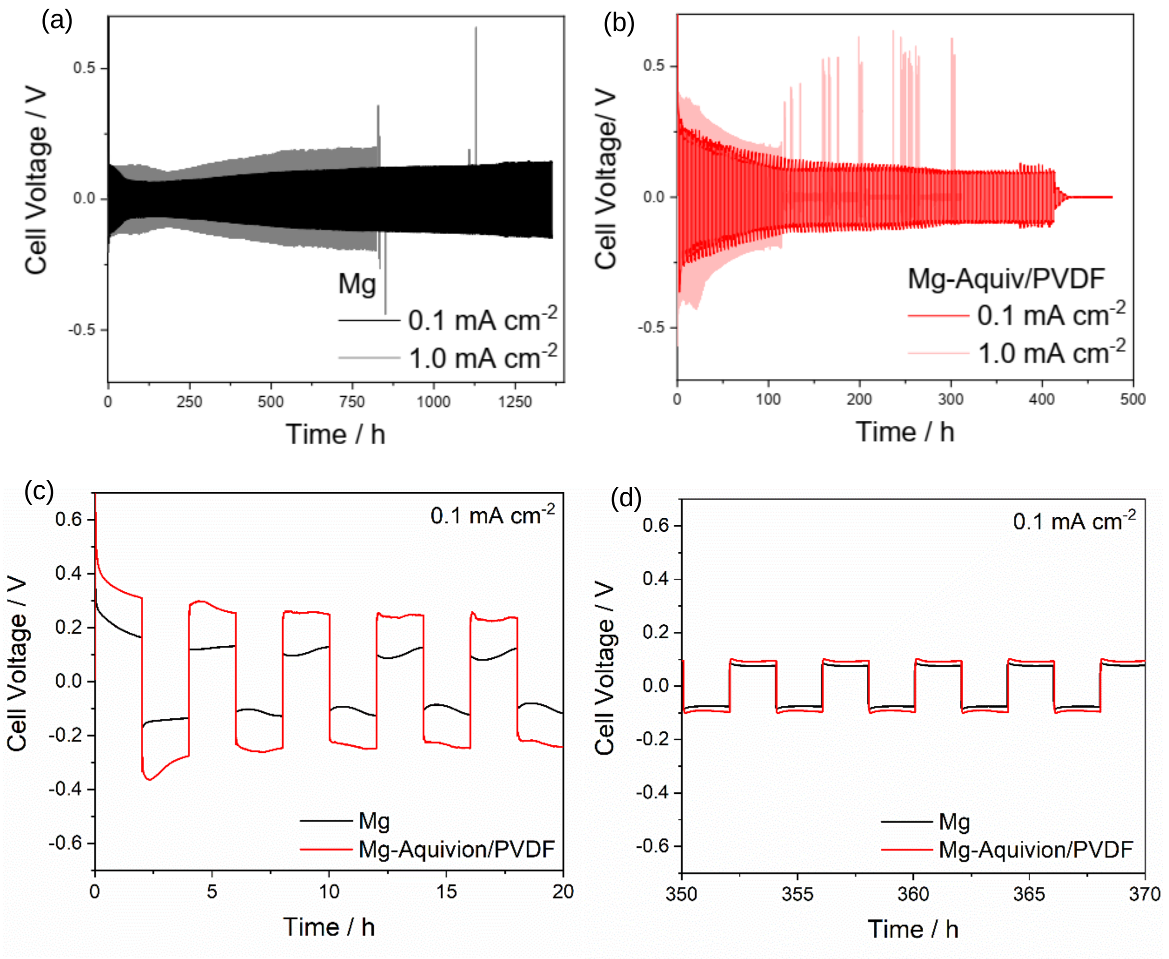

Figure S8: Long-term polarization of Mg-Mg cells comprising (a) pristine Mg and (b) coated Mg at 0.1 and 1.0 mA cm^{-2}. (c+d) Direct comparison of the polarization overpotential at 0.1 mA cm^{-2}.

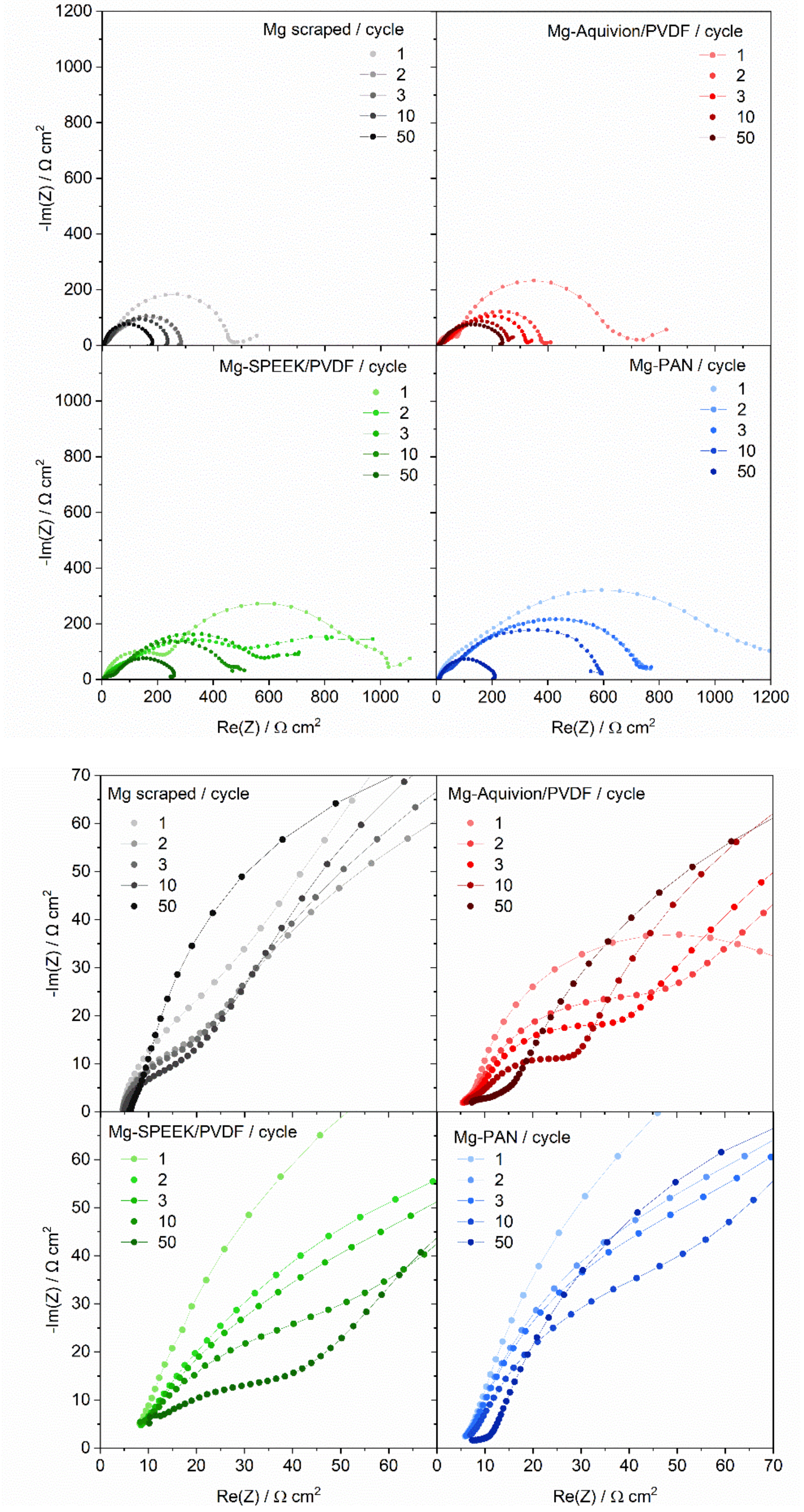

Figure S9: Nyquist plots of impedance spectra of pristine and coated Mg anodes during stripping at 0.1 mA cm^{-2} (Bottom: zoomed high-frequency region). The corresponding polarization cycles are depicted in Figure 4c and g in the manuscript.

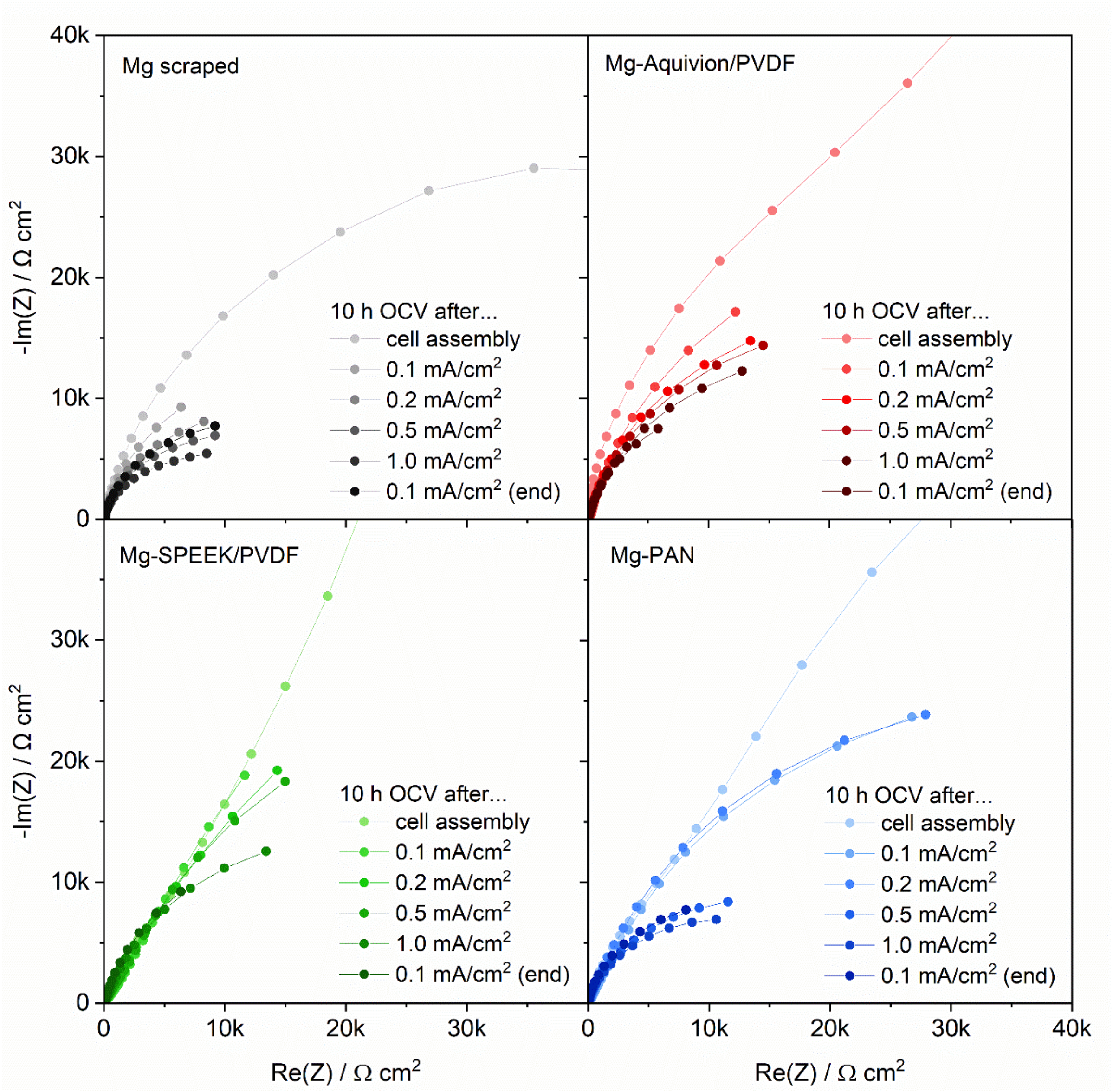

Figure 10: Nyquist plots of impedance spectra of pristine and coated Mg anodes 10 h after cell assembly and polarization, respectively. The measurement point corresponds to 10 h, 107h, 142 h, 165 h, 185 h, 245 h in Figure 4a in the manuscript.

Ante and post mortem analysis of pristine and coated Mg anodes

The EDX analysis of the pristine and coated Mg anodes is depicted in Table S1. Therein, small detected amounts of Na, Al and Si (below 0.5 at.%) in some post mortem samples due to separator fiber residues were not listed.

Table S1: EDX analysis of pristine and coated Mg anodes prior and after cycling.

	Sample	Image	Position	wt.% C	O	F	Mg	S
**Bare Mg**	after scraping	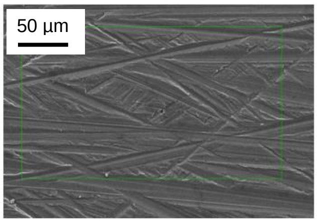	1	2.43	3.26	-	94.31	-
	post mortem	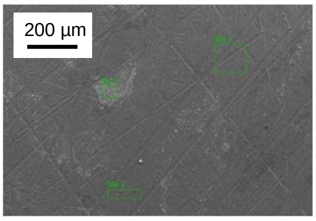	1	2.67	2.15	-	95.18	-
			2	2.83	1.62	-	95.55	-
			3	4.30	7.49	0.82	80.88	6.51
	oxidized	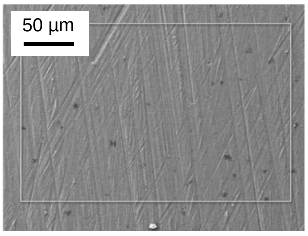	1	2.72	1.06	-	96.23	-
	post mortem	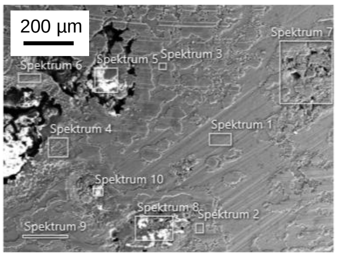	1	4.50	2.01	0.37	93.12	-
			2	4.87	3.26	0.89	90.65	0.34
			3	4.97	12.87	2.47	78.11	1.57
			4	7.70	8.12	1.81	81.85	0.52
			5	10.20	29.96	8.19	49.17	2.31
			6	6.17	6.13	1.01	86.41	0.28
			7	7.33	11.53	2.45	77.82	0.88
			8	8.80	30.24	6.03	51.31	3.63
			9	4.00	3.49	0.79	91.45	0.28
			10	10.49	36.42	11.68	38.29	2.84
**Mg-Aquivion/PVDF**	after spin coating	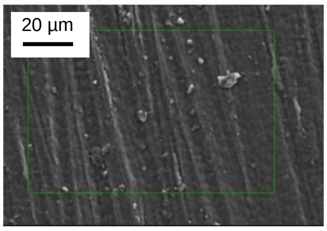	1	26.33	5.91	23.09	43.97	0.72
	post mortem	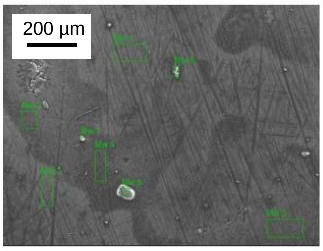	1	16.17	8.46	10.78	64.58	-
			2	14.74	8.12	9.53	67.61	-
			3	16.18	13.34	14.34	54.56	0.67
			4	16.76	15.95	16.42	49.19	0.71
			5	16.28	14.20	13.80	54.16	0.61
			6	9.40	5.92	4.35	80.34	-
			7	13.61	9.43	10.94	64.92	0.57
			8	19.81	22.24	29.19	23.62	5.15

	after tape casting	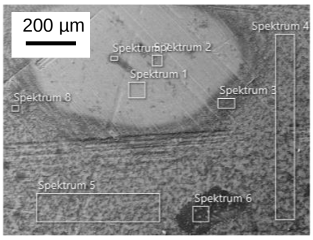	1	4.51	1.98	0.61	92.91	-
			2	8.76	16.76	1.31	71.85	0.25
			3	21.06	3.89	15.31	59.02	0.72
			4	21.52	4.79	15.23	57.93	0.53
			5	21.93	5.82	15.96	55.71	0.58
			6	25.19	7.77	36.31	29.09	1.65
			7	16.65	2.97	8.95	71.05	0.37
			8	20.14	4.63	18.31	56.01	0.92
	post mortem	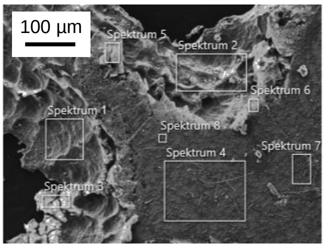	1	8.84	13.91	2.56	73.34	0.85
			2	9.37	32.87	7.43	47.43	2.51
			3	11.96	38.45	10.90	35.98	2.40
			4	23.92	6.63	21.16	43.80	1.39
			5	27.54	14.37	38.11	17.61	1.80
			6	20.40	12.06	19.60	43.63	2.18
			7	24.55	11.83	26.35	33.88	2.04
			8	27.31	5.22	21.44	40.00	1.74
**Mg-SPEEK/PVDF**	after spin coating	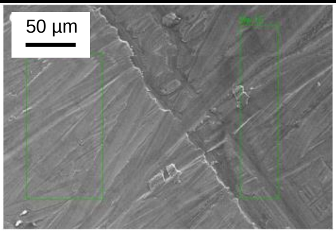	14	12.95	10.23	2.44	73.73	0.65
			15	30.15	7.72	7.49	53.26	1.38
	post mortem	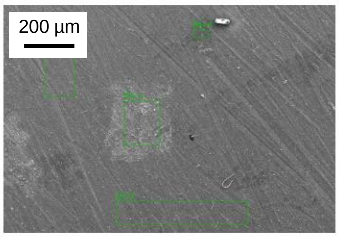	1	2.28	1.63	-	96.10	-
			2	2.63	2.23	-	95.15	-
			3	4.26	4.33	-	86.95	4.46
			4	2.65	20.37	-	75.33	1.64
**Mg-PAN**	after spin coating		not determinable					
	post mortem	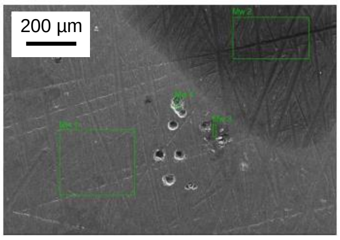	1	3.43	3.09	-	93.47	-
			2	34.69	4.40	2.41	48.96	1.78
			3	5.37	35.84	3.28	52.86	2.65
			4	4.93	5.32	0.66	89.09	-

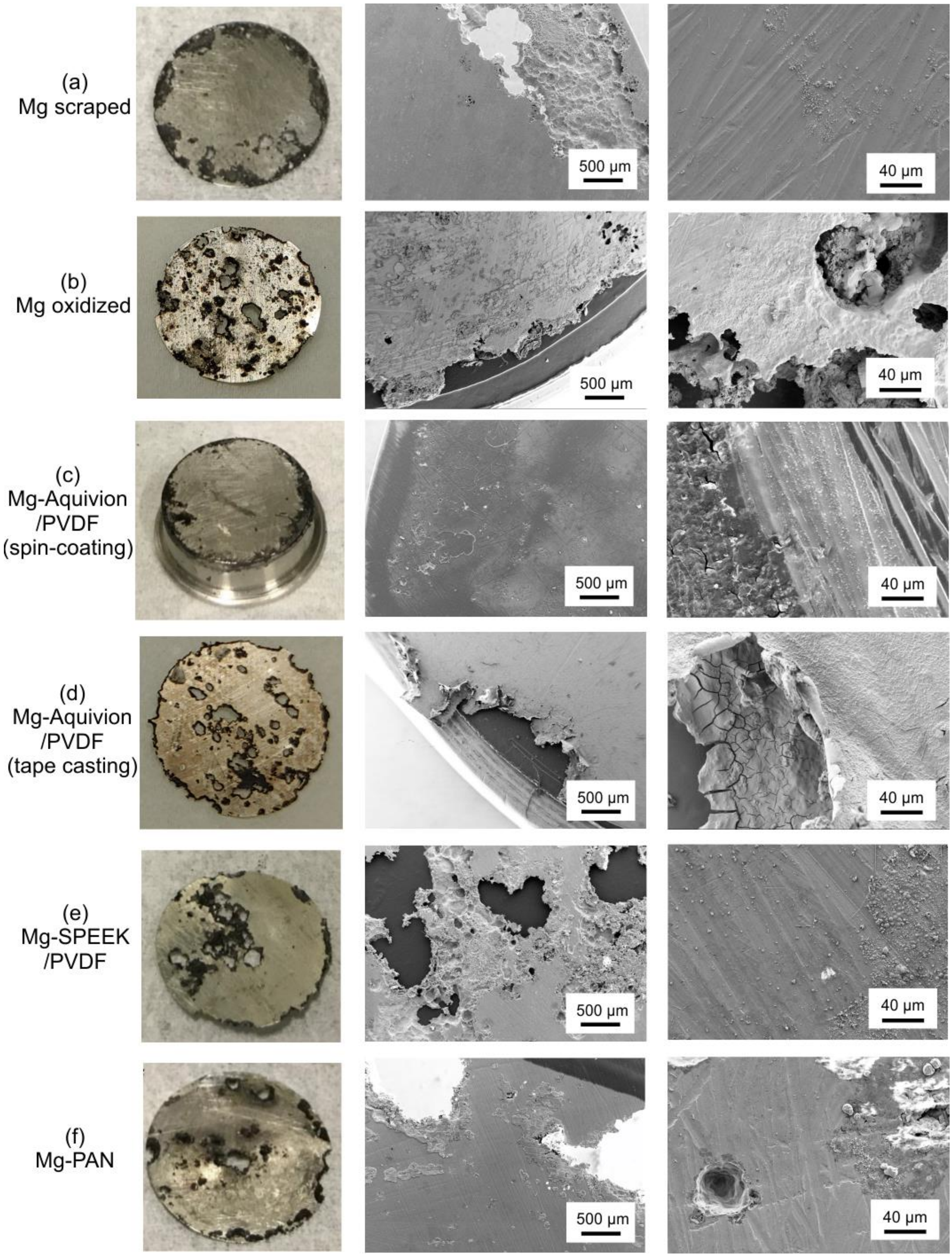

Figure S11: Post mortem photo and SEM images of pristine and coated Mg anodes after cycling at C/10 in Mg-S cells.

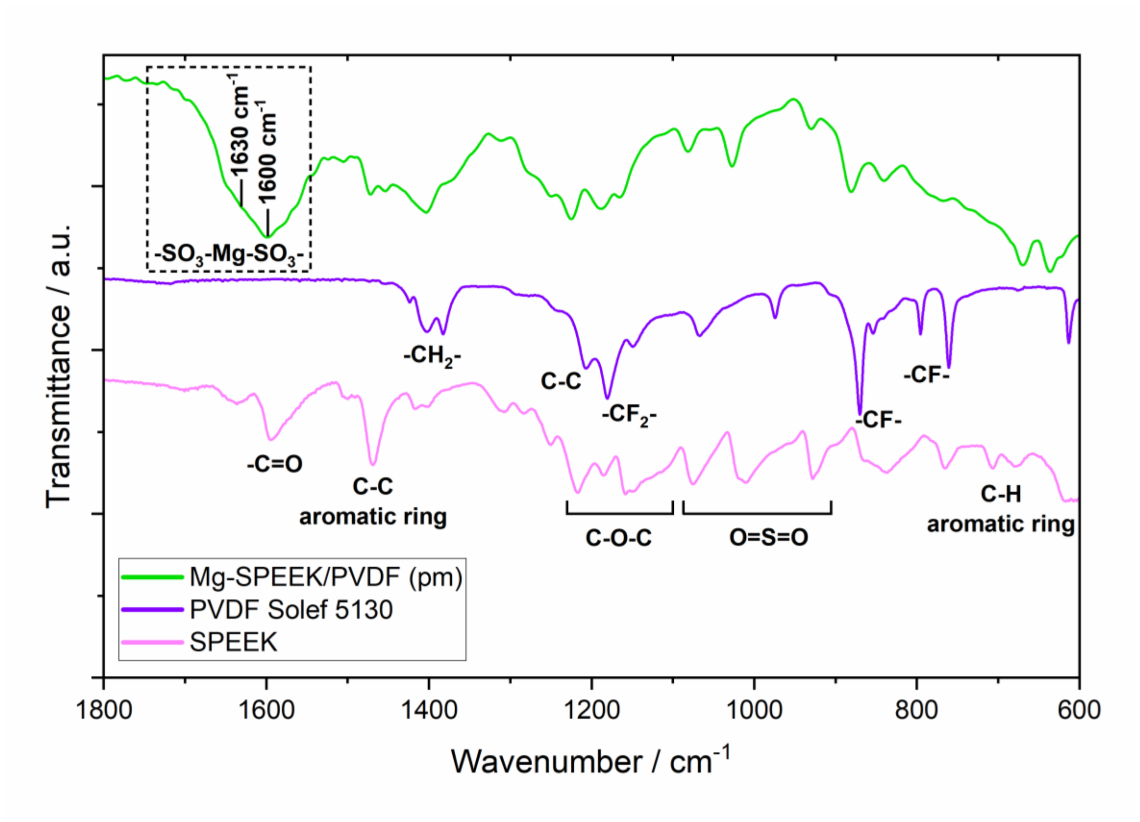

Figure S12: FTIR spectra of a Mg-SPEEK/PVDF anode (Mg-S cell, post mortem) in comparison to PVDF and SPEEK powder. In situ cation exchange and consequent crosslinking of the SO_3^- groups are likely due to the strong absorbance at approx. 1600 cm^{-1} and 1630 cm^{-1}.

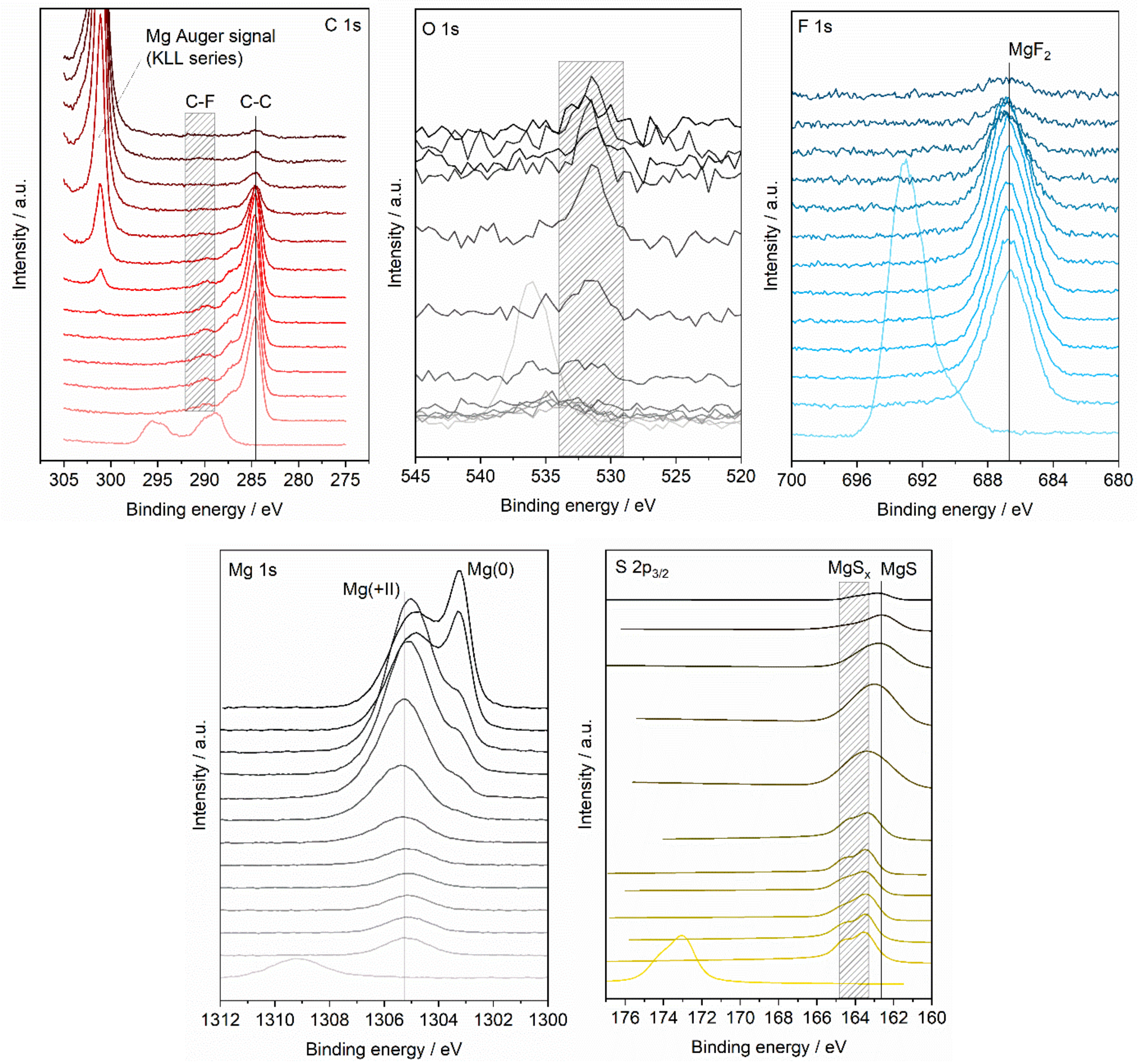

Figure S13: Photoelectron spectra of C 1s, O 1s, F 1s, Mg 1s and S 2p with the proposed compounds due to the peak binding energy. The bottom spectra (before ion etching) are each shifted by approx. 4 eV due to local charging. While the Mg 1s signal can be clearly attributed to Mg(0) and Mg(+II), the predominant fluorine signal matches a metal fluoride, and not an organic fluorine component. The S 2p spectra show the gradual shift from a polysulfide to a monosulfide, when getting in close contact to magnesium. The C 1s spectra only show a low $C\text{-}F_2$ signal (291-292 eV) – either due to electrochemical processes or the XPS analysis with focused X-ray and ion beams itself. The O 1s signals (recorded with a lower signal quality) cannot be attributed to a specific compound by their spectral position and appear at the beginning of the metallic magnesium substrate.

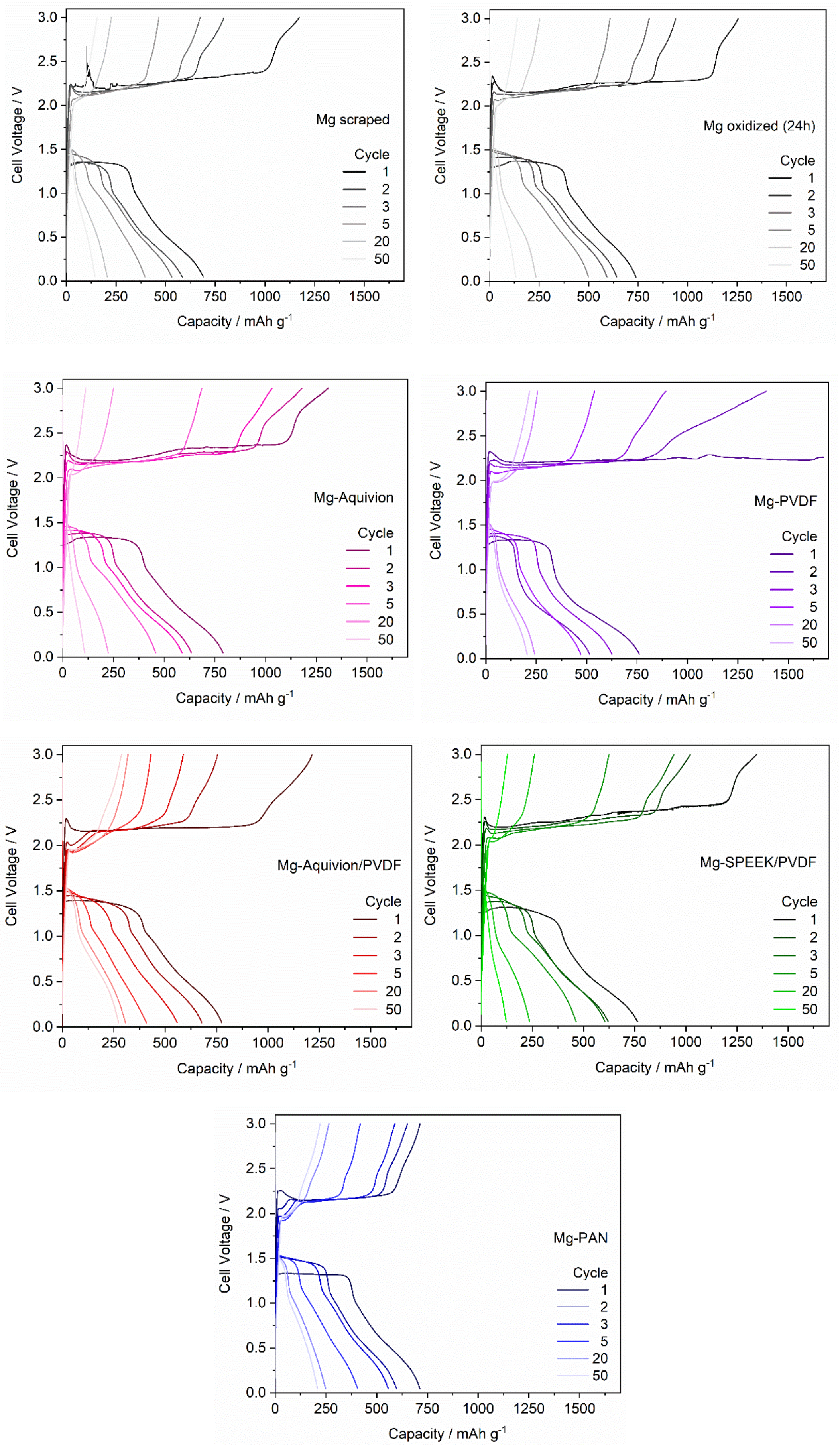

Figure S14: Discharge and charge cycles of Mg-S cells (C/10) with scraped, oxidized and spin-coated Mg anodes.

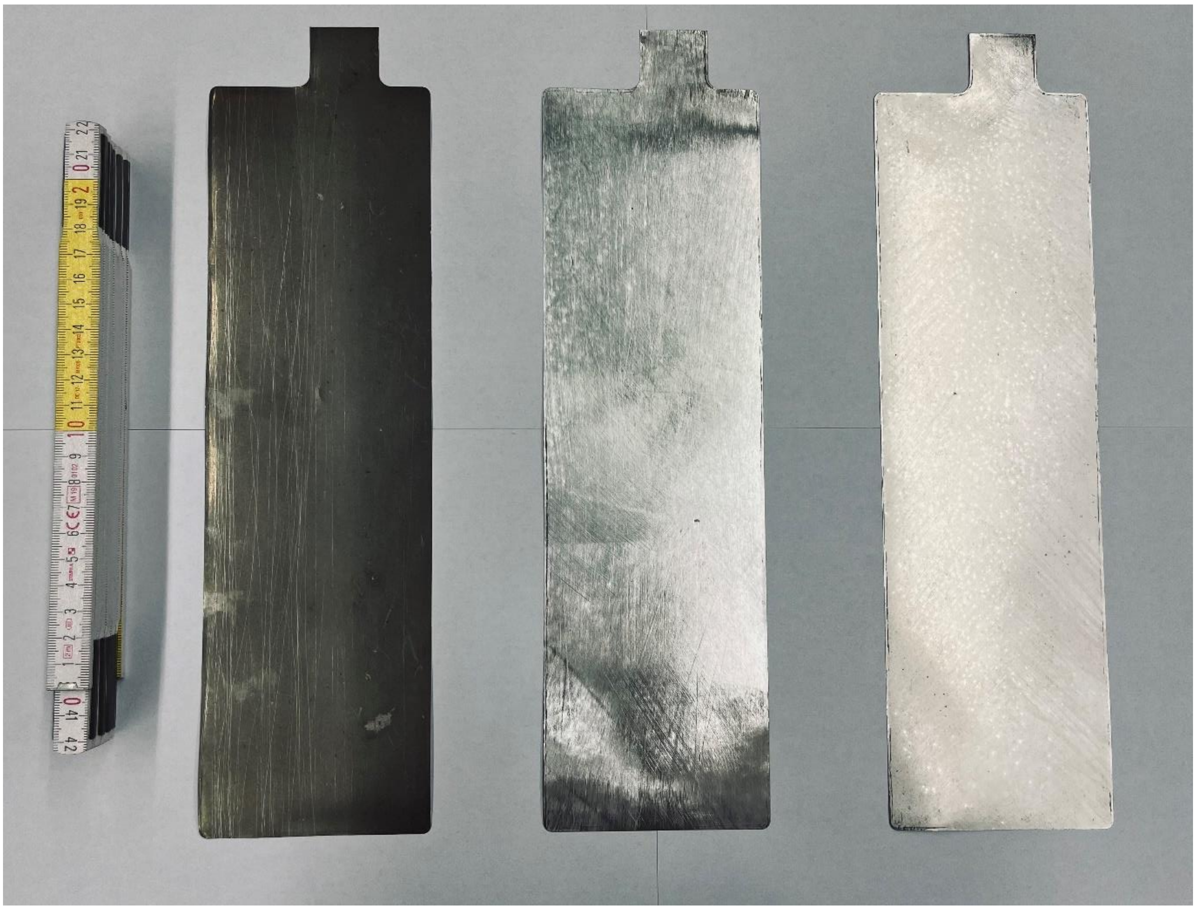

Figure S15: Mg electrodes sheets in large format (320 x 95 mm without tab, 0.1 mm thickness): As received (left), scrapped with a glass plate (middle) and coated with an Aquivion/PVDF layer (via tape casting, approx. 1 µm thickness, right).

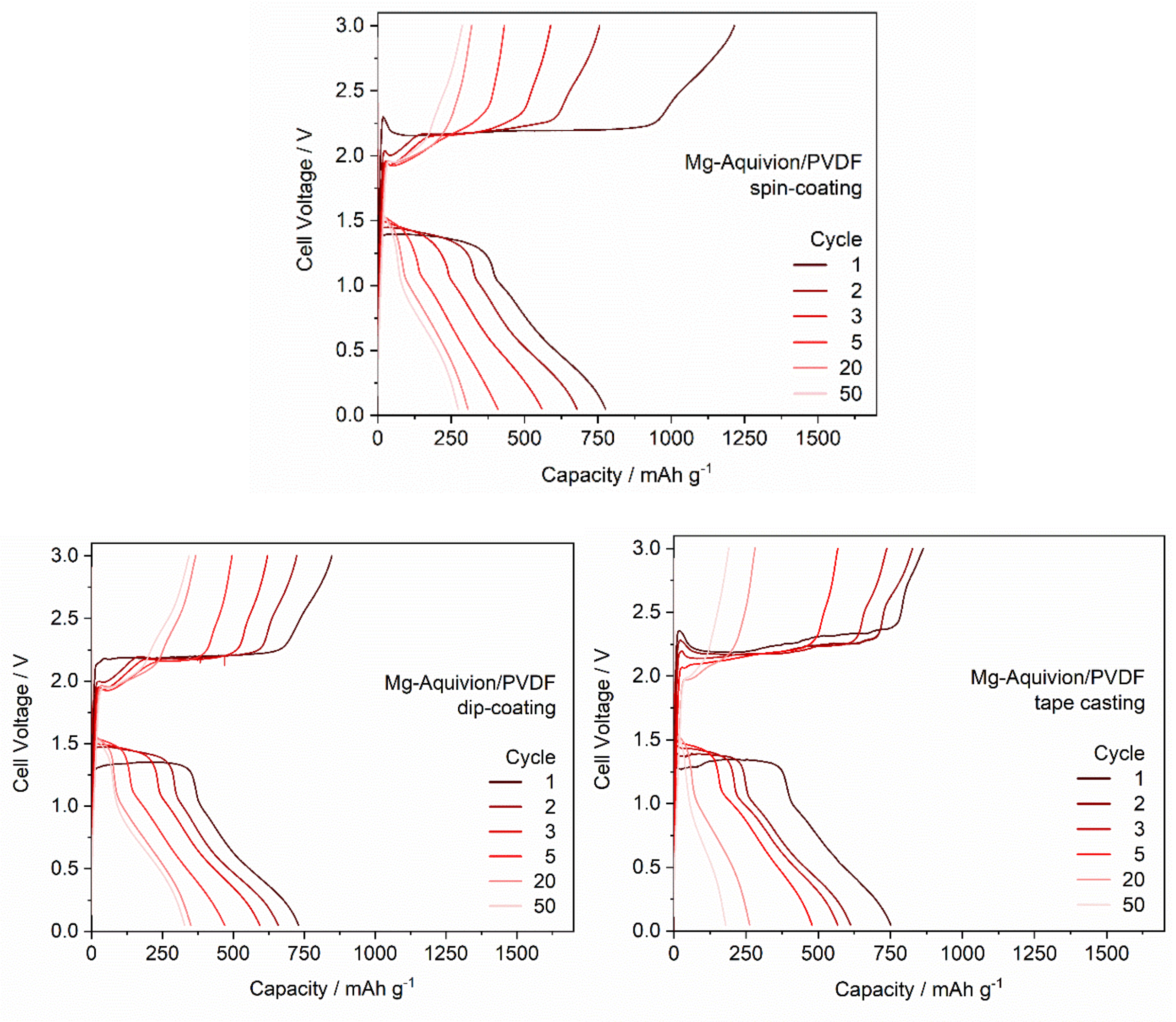

Figure S16: Discharge and charge cycles of Mg-S cells (C/10) with Aquivion/PVDF coated Mg anodes prepared via spin-coating, dip-coating (red) and tape casting (yellow).
